# Improving sustainable isopropanol production in engineered *Escherichia coli* W via oxygen limitation

**DOI:** 10.1186/s12934-025-02720-1

**Published:** 2025-04-26

**Authors:** Regina Kutscha, Dominic Uhlir, Stefan Pflügl

**Affiliations:** Institute of Chemical, Environmental and Bioscience Engineering, Technische Universität Wien, Gumpendorfer Straße 1a, Vienna, 1060 Austria

**Keywords:** Microaerobic conditions, Oxygen limitation, *Escherichia coli*, Isopropanol, Lactose, Whey, Sustainable bioprocessing

## Abstract

**Background:**

Due to ecological concerns, alternative supply lines for fuel and bulk chemicals such as isopropanol are increasingly pursued. By implementing the formation pathways from natural producers like *Clostridium beijerinckii* and *Clostridium aurantibutyricum*, isopropanol can be produced in *Escherichia coli*. However, developing an industrially and economically feasible microbial production process requires a robust and efficient process strategy. Therefore, this study explores microaerobic conditions in combination with lactose and sour whey as sustainable carbon source as a basis for large-scale microbial isopropanol production.

**Results:**

Different gas-liquid mass transfer regimes (affected by variations of the stirrer speed and ingas oxygen concentration) allowed the implementation of different microaerobic conditions characterized by their specific oxygen uptake rate (q_O2_) in cultivations with an isopropanol producing *E. coli* W strain on lactose. Under microaerobic conditions, the specific isopropanol production rate (q_p, ipa_) exhibited a direct correlation with q_O2_. Moreover, isopropanol production showed a pseudo growth-coupled behavior. Monitoring of the formation rates of various by-products such as acetone, lactate, acetate, pyruvate, formate and succinate allowed to identify a q_O2_ of 9.6 mmol g^− 1^ h^− 1^ in only slightly microaerobic cultivations as the best conditions for microbial isopropanol production. Additionally, the data suggests that a carbon bottleneck exists at the pyruvate node and the availability of the redox factor NADPH is crucial to shift the product balance from acetone to isopropanol. Finally, confirmation runs prove the effectiveness of the microaerobic production approach by yielding 8.2 g L^− 1^ (135.8 ± 13.3 mmol L^− 1^) and 20.6 g L^− 1^ (342.9 ± 0.4 mmol L^− 1^) isopropanol on lactose and whey, respectively, reaching a volumetric isopropanol formation rate of up to 2.44 g L^− 1^ h^− 1^ (40.6 mmol L^− 1^ h^− 1^).

**Conclusions:**

This study identifies slightly microaerobic conditions (q_O2_ ~ 10 mmol g^− 1^ h^− 1^) as the currently best conditions for microbial isopropanol production on lactose and whey in *E. coli* W. In the future, optimizing the carbon flux around the pyruvate node, ensuring sufficient NADPH supply, and establishing a feedback control loop to control process variables affecting oxygen transfer to the culture, could make microbial isopropanol production feasible at an industrial scale.

**Supplementary Information:**

The online version contains supplementary material available at 10.1186/s12934-025-02720-1.

## Background

Due to growing concerns about dependencies on fossil fuels and associated greenhouse gas emissions, there is an increasing interest in alternative supply lines for fuels and bulk chemicals [1]. In this regard, isopropanol is of considerable importance. Its applications are diverse and include disinfectants, degreasers, additives to cosmetics and pharmaceutical products, fuel additives, chemical extractions and rubber manufacturing [2–4]. On an industrial scale, isopropanol is currently produced via direct or indirect hydration of propylene obtained from steam cracking [5, 6]. In contrast to this, establishing a microbial production can serve as a more ecologically friendly supply line [2, 7–10].

**Fig. 1:**
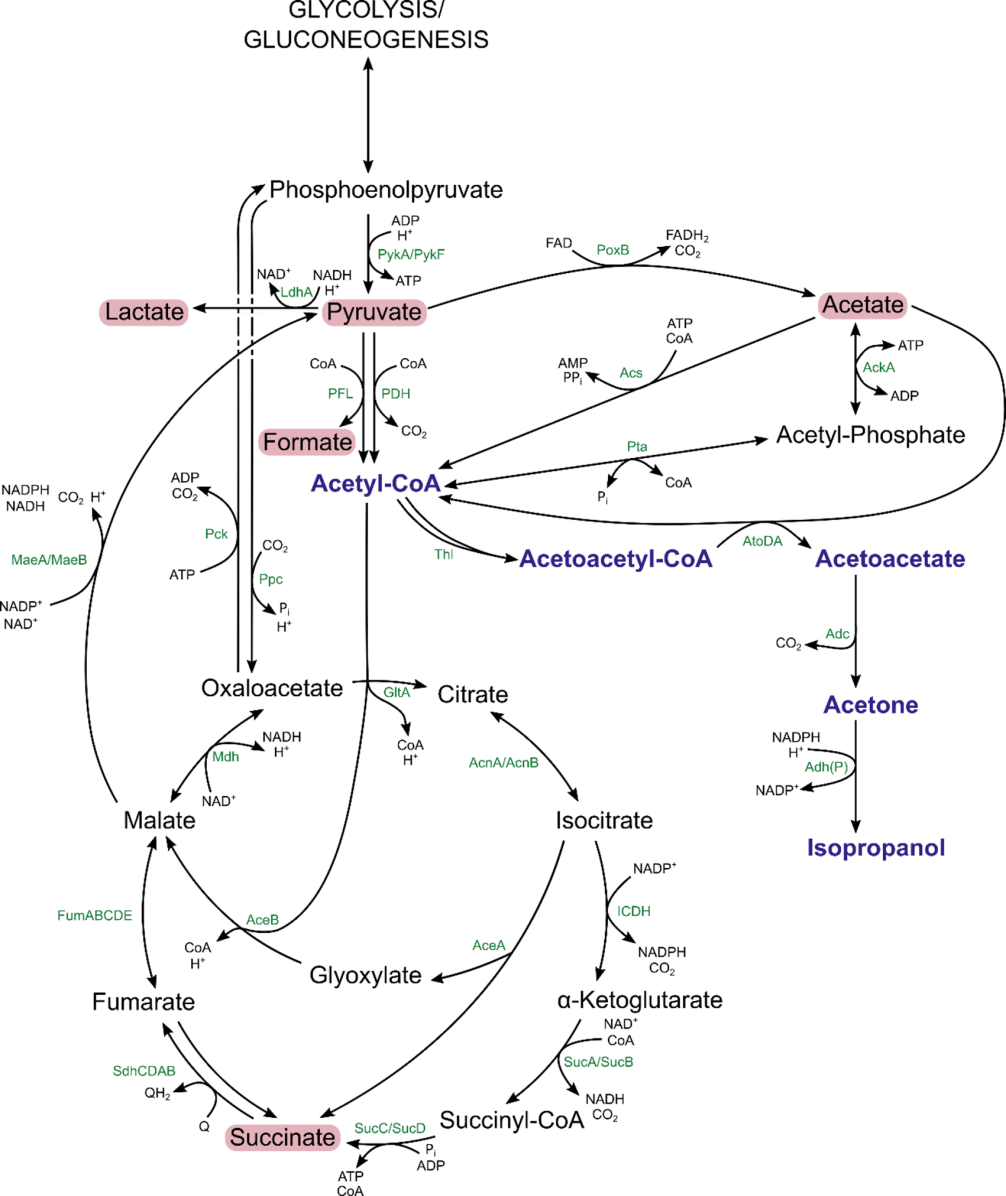
Metabolic network around the pyruvate and acetyl-CoA nodes in *E. coli* W engineered for isopropanol production

Despite *Clostridium beijerinckii* and *Clostridium aurantibutyricum* being natural producers of isopropanol, various engineered production hosts have already been screened [11]. In the frequently used *Escherichia coli* a heterologous pathway has to be introduced to enable the conversion of acetyl-CoA to acetone and isopropanol as shown in Fig. 1 [8]. Since the isopropanol pathway branches off at the heart of the central carbon metabolism, balancing the carbon flux between biomass growth and isopropanol formation has been identified as a crucial way to improve production. Consequently, previous research has focused on the metabolic engineering of the acetyl-CoA and pyruvate node [12, 13].

An alternative method to shift between growth and production is the implementation of a nutrient limitation [14, 15]. In contrast to metabolic engineering approaches, nutrient limitations constitute operational strategies that are not routinely combined with metabolic engineering in *E. coli* [16]. Possible limitation targets range from carbon, nitrogen, phosphorous, iron, oxygen and sulfur to potassium and magnesium.

Microaerobic or oxygen-limiting conditions have been implemented for some time in order to improve the formation of fermentative products in *E. coli* [17–21]. Extensive studies have revealed a complex regulatory process behind the switch from aerobic to anaerobic conditions [22]. These highly dynamic conditions pose two challenges for applications in industrial bioprocess scenarios. On the one hand, microaerobic conditions are achieved only in the dynamic transition between aerobic and anaerobic conditions, entailing potentially changing carbon flows in the cells. On the other hand, the possibilities to exactly define microaerobic conditions in a bioreactor setting are severely limited, since commercially available oxygen probes are unable to measure such low dissolved oxygen concentrations. Oxygen-limitation can also exacerbate undesired side product formation [23]. However, recent findings suggest the favorable combination of microaerobic conditions with certain carbon sources (lactose) [24].

The choice of carbon source is vital to establish a sustainable bioprocess. Recently, microbial production from renewable carbon sources and waste streams has sparked increasing interest [25–28]. Cheese whey constitutes one of these waste stream-derived carbon sources. Originating from ultrafiltration of whey protein isolates, whey valorization reduces costs for waste disposal [29]. Cheese whey requires less pre-processing and contains less inhibitory or toxic compounds than other renewable carbohydrate-containing feedstocks such as lignocellulosic hydrolysates and is therefore more easily applicable in bioprocesses [30].

Combining all necessary requirements, *E. coli* W is a strong candidate for attempting sustainable microbial isopropanol production. The firmly established genetic engineering tools allow for fast recombinant pathway introduction and further modifications to balance growth and production [8, 9, 12, 13]. In addition, its capabilities to metabolize lactose together with the favorable lactose-oxygen-limitation approach are predestined for sustainable bioprocess development [24].

Therefore, the goal of this study was the production of isopropanol from whey/lactose. We first wanted to determine whether knockouts of fermentative pathways (∆*ldhA*, ∆*adhE*, ∆*pta* and ∆*frdA*) could improve recombinant isopropanol production in an *E. coli* W host strain. Next, a suitable bioprocessing strategy needed to be established. For balancing growth and production, oxygen-limitation served as the tool of choice. The extent of the necessary limitation was determined in a Design of Experiments (DoE) approach and led to insights into the central carbon metabolism of the engineered strain. Application of these insights resulted in an improved process for isopropanol synthesis from lactose, both in synthetic medium as well as in whey from sour whey.

## Materials and methods

### Bacterial strains and media

*E. coli* W (DSM 1116 = ATCC 9637 = NCIMB 8666) from DSMZ (Braunschweig, Germany), *E. coli* W Δ*ldhA* Δ*adhE* (KO2) and *E. coli* W Δ*ldhA* Δ*adhE* Δ*pta* Δ*frdA* (KO4) (kind gifts from Prof. Michael Sauer, BOKU, Vienna, Austria) were used as host organisms.

For all cloning, plasmid propagation steps and first precultures liquid lysogeny broth (LB) prepared with 5 g L^− 1^ yeast extract, 10 g L^− 1^ soy peptone, 10 g L^− 1^ NaCl and 15 g L^− 1^ of agar agar (for plates only), containing the appropriate amount of antibiotic was used.

The base medium (used at pH 7) for all further cultivation steps was derived from Riesenberg et al. [31]. The medium contained: 13.3 g L^− 1^ KH_2_PO_4_, 4.0 g L^− 1^ (NH_4_)_2_HPO_4_,1.7 g L^− 1^ citric acid (autoclaved), 1.2 g L^− 1^ MgSO_4_ * 7 H_2_O, 0.10 g L^− 1^ Fe(III)citrate, 0.0084 g L^− 1^ EDTA, 0.013 g L^− 1^ Zn(CH_3_COO)_2_ * 2 H_2_O, 0.0025 g L^− 1^ CoCl_2_ * 6 H_2_O (Merck KGaA, Darmstadt, Germany), 0.015 g L^− 1^ MnCl_2_ * 4 H_2_O, 0.0012 g L^− 1^ CuCl_2_ * 2 H_2_O, 0.0030 g L^− 1^ H_3_BO_3_, 0.0025 g L^− 1^ Na_2_MoO_4_ * 2 H_2_O (sterile filtered). For the second preculture step, 10 g L^− 1^ of glucose were used as carbon source. The shake flask main cultures contained 10 g L^− 1^ of lactose. All media were supplied with 50 mg L^− 1^ of kanamycin.

Concentrated sour whey (25% dry mass) was kindly provided by NÖM AG (Niederösterreichische Molkerei AG, Baden, Austria). Frozen stocks of sour whey concentrate were thawed and heated to 100 °C for 30 min to precipitate lipids and proteins. After centrifugation for 30 min at 15,000 g and 20 °C, the sour whey concentrate was filtered via tangential flow filtration using 0.22 μm membrane cassettes (Centramate™, PALL Life Sciences, Port Washington, NY, USA). To sterilize the permeate, it was again filtered via sterile 0.22 μm bottle top filters into sterile glass vessels.

For bioreactor cultivations with whey, sterile concentrated sour whey was added to the base medium to reach a lactose concentration of approximately 88 mmol L^− 1^ (30 g L^− 1^) at the start of the batch phase. For bioreactor cultivations with lactose, the batch medium contained 146 mmol L^− 1^ (50 g L^− 1^) lactose. The feed/pulse medium contained 438 mmol L^− 1^ (150 g L^− 1^) lactose and thrice the amount of MgSO_4_ * 7 H_2_O, Fe(III)citrate, EDTA, Zn(CH_3_COO)_2_ * 2 H_2_O, CoCl_2_ * 6 H_2_O, MnCl_2_ * 4 H_2_O, CuCl_2_ * 2 H_2_O, H_3_BO_3_ and Na_2_MoO_4_ * 2 H_2_O present in the batch medium to avoid undesired nutrient depletion. If whey was used for the feed/pulse medium, it also contained 83–111 mmol L^− 1^ (15–20 g L^− 1^) of galactose and 133–167 mmol L^− 1^ (12–15 g L^− 1^) lactate.

### Strain engineering

Engineering of the *E. coli* strains (*E. coli* W_IPA, *E. coli* W KO2_IPA and *E. coli* W KO4_IPA) for isopropanol production was described in a previous study [32]. Briefly, the genes *thl*, *adc* (*Clostridium acetobutylicum*), and *adh* (*C. beijerinckii*) were codon optimized and together with *atoDA* (amplified from *E. coli* MG1655) assembled into a pathway with constitutive promoters via Golden Gate cloning. Transferring the plasmid with the pathway genes to the different host strains (see Sect. 2.1) enabled isopropanol production.

### Preculture preparation

Frozen stocks of the production strain were kept at -80 °C in 20% (w/v) glycerol. To start cultivations, cells were streaked onto LB-plates containing 50 mg L^− 1^ kanamycin and incubated overnight at 37 °C. Afterwards, single colonies were picked to inoculate the first preculture step on 50 mL of LB in 500 mL shake flasks. These flasks were incubated for approximately 8 h at 37 °C and 230 rpm. After checking the optical density at 600 nm (OD_600_) of the cells, the second preculture step was inoculated to a starting OD_600_ of 0.05. The cells then grew for about 16 h at 37 °C and 230 rpm in 50 mL (in 500 mL shake flasks for shake flask experiments) or 200 mL (in 2 L ultra-high yield flasks for bioreactor cultivations) of defined preculture medium. To avoid transferring carbon source to the main culture, the cells were centrifuged at 4000 g for 5 min, resuspended in sterile 0.9% (w/v) NaCl solution in 25% of the preculture volume, centrifuged again at 4,000 g for 5 min and resuspended in 5 mL (shake flasks) or 10 mL (bioreactors) of sterile 0.9% (w/v) NaCl solution. The optical density was determined, and the main cultures were inoculated to an OD_600_ of 0.5.

### Shake flask cultivations

The cells grew for 48 h at 37 °C and 230 rpm. 1 mL samples were taken regularly to determine OD_600_ and metabolite concentrations via HPLC measurement.

### Bioreactor cultivations

Bioreactor cultivations were carried out in four parallel DASGIP^®^ Benchtop Bioreactors for Microbiology (Eppendorf AG, Hamburg, Germany) with 1 L working volume. The temperature was kept at 37 °C. The pH was monitored by an EasyFerm Plus PHI K8 225 (Hamilton, Reno, NV, USA) electrode and maintained at 7 by adding 12.5% (v/v) of NH_3_ or 5 M H_3_PO_4_ via a MP8 multi-pump module (Eppendorf AG, Hamburg, Germany). Dissolved oxygen concentration was measured with a VisiFerm DO ECS 225 H2 probe (Hamilton, Reno, NV, USA) and kept at 30% for aerobic runs by increasing stirrer speed (up to 1,000 rpm). An exponential feeding profile was carried out for the aerobic cultivations with a target specific growth rate of 0.1 h^− 1^. The gassing rate was 60 L h^− 1^ (1 vvm) for all cultivations. The off gas was led through three consecutive wash bottles filled with 400 mL dH_2_O on ice before being analyzed for O_2_ and CO_2_ by a GA4 gas analyzer module (Eppendorf AG, Hamburg, Germany). Samples were taken regularly from the reactors and all wash flasks. Biomass formation was estimated by measuring OD_600_ in a spectrophotometer (Onda Spectrophotometer V-10 Plus, Giorgio Bormac s.r.l, Carpi (MO), Italy). After centrifugation of the cultivation broth (21,913 g, 10 min, 4 °C), concentrations of lactose, galactose, lactate, acetate, pyruvate, formate, succinate, ethanol, acetone, and isopropanol were determined via HPLC measurement in the supernatant.

### Microaerobic design of experiments

To generate a microaerobic environment based on stirrer speed and oxygen concentration in the ingas a design space with three stirrer speeds (400, 700 and 1,000 rpm) and three ingas concentrations (10, 20 and 30% oxygen) in a full factorial central composite face design with a triplicate center point was set up. Cultivations were carried out in pulsed batch mode with one pulse containing 250 mL of pulse medium. We defined microaerobic conditions as conditions in which measurable dissolved oxygen concentration was zero. Depending on the stirrer speed and ingas concentrations these conditions were reached at different times and different biomass concentrations. This allowed for classification into severely limited conditions (early onset of limitation and long duration) and slightly limited conditions (late onset of limitation and short duration).

### Biomass determination

Dry cell weight (DCW) was determined gravimetrically in triplicates. 1 mL of culture broth was centrifuged in pre-weighed 2 mL Eppendorf tubes at 21,913 g for 10 min at 4 °C. Next, 1 mL of filtered 0.9% (w/v) NaCl solution served to resuspend the cells. They were again centrifuged, the supernatant discarded and the cells dried at 110 °C for 72 h. A correlation between OD_600_ and DCW was established to estimate DCW for very low biomass concentrations.

### HPLC analysis

Alcohols and organic acids were determined on an Aminex HPX-87 H column (300 × 7.8 mm, Bio-Rad, Hercules/CA, USA) in a Vanquish™ Core HPLC System (Thermo Scientific, Waltham/MA, USA). The mobile phase was 4 mM H_2_SO_4_, the flow rate set to 0.6 mL min^-^^1^ and the method was run at 60 °C for 30 min. Cultivation broth supernatants were diluted 1:10 before sample preparation. Wash flask samples were measured undiluted. The injection volume was 10 µL. Metabolites were detected via a refractive index detector (ERC RefractoMax 520, Thermo Scientific, Waltham/MA, USA) and an UV detector (Vanquish™ Variable Wavelength Detector Vanquish VWD-C, Thermo Scientific, Waltham/MA, USA) at 280 nm (acetone and pyruvate).

The 540 µL of (diluted) sample were mixed with 60 µL of 40 mM H_2_SO_4_ and centrifuged at 21,913 g for 10 min at 4 °C. The supernatant was used for analysis. Standards were treated the same as the samples and a 6-point calibration curve was used for quantification between 0.05 and 10 g L^− 1^.

### DoE - statistical data analysis

Overall yields and specific rates were calculated for the microaerobic conditions in the DoE cultivations and used as responses for the quadratic model. The model design and the analysis were set up and performed in the MODDE^®^ Pro 13 software (Sartorius AG, Göttingen, Germany). Initially, the full quadratic model was used for all responses, but insignificant model terms were removed after careful inspection of the coefficient plots. Since the whole model was subject to biological variation, terms that were only very slightly insignificant were not removed. Optimum conditions were defined as having very few side products and high product yields. The results were visualized in contour and coefficient plots (cf. to Supplementary Fig. 1–10, Supplementary Material 1).

## Results and discussion

### Preliminary strain screening

To test the isopropanol production capability of the three generated *E. coli* strains (*E. coli* W_IPA, *E. coli* W KO2_IPA and *E. coli* W KO4_IPA), we conducted shake flask experiments on lactose. The strains used in this study were constructed in a previous study to establish an efficient isopropanol production on acetate [32]. For a statistical analysis of the results please cf. to Supplementary Tables 6–14 in Supplementary Material 1.

**Fig. 2:**
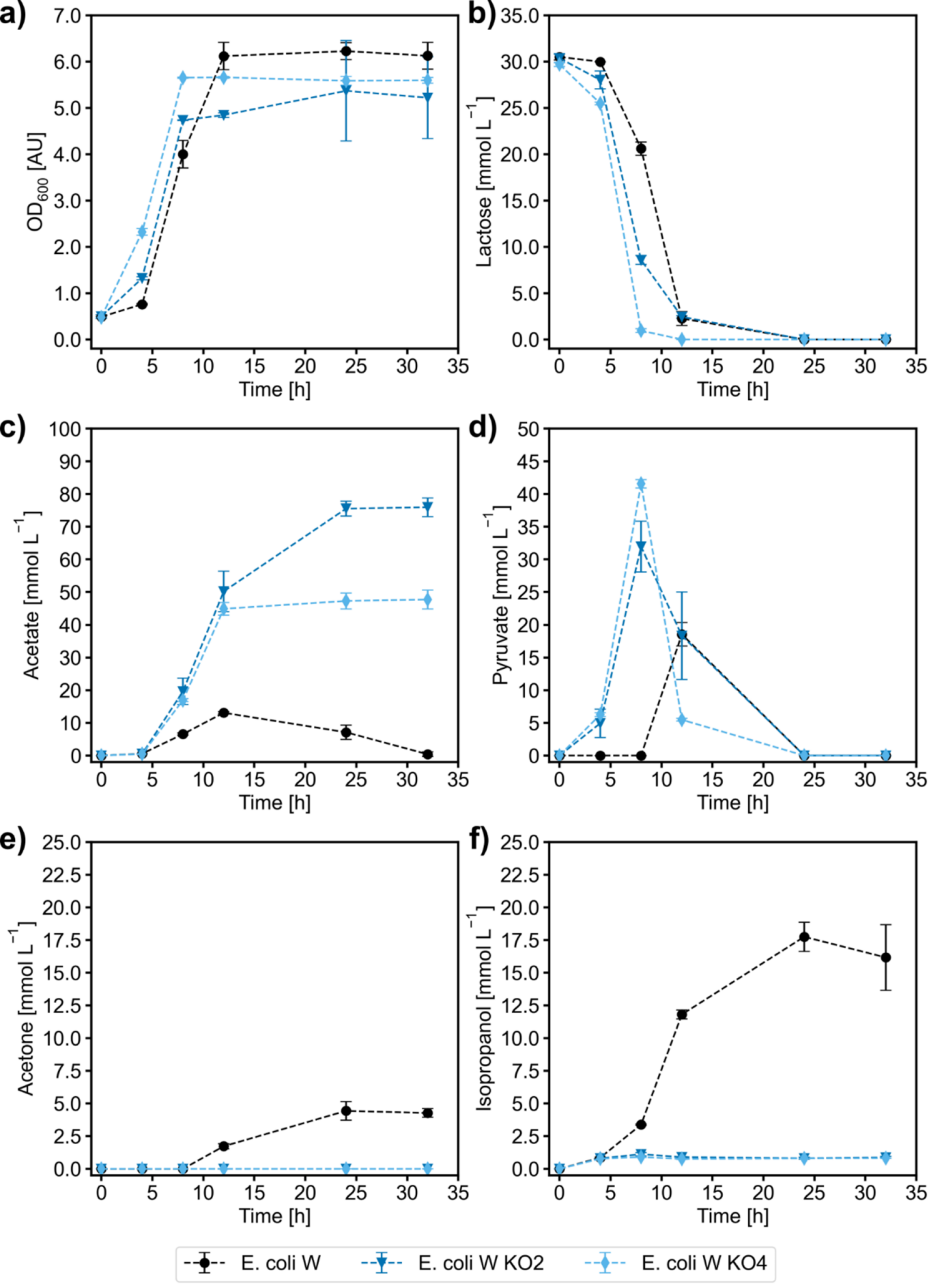
Shake flask experiments on lactose with isopropanol producing *E. coli* W, *E. coli* KO2 and *E. coli* KO4. Profiles of OD_600_ (**a**), lactose (**b**), acetate (**c**), pyruvate (**d**), acetone (**e**) and isopropanol (**f**) are shown as means of independent biological triplicates

All strains grew with a comparable specific growth rate (0.27–0.29 h^− 1^) in the first 8 h of cultivation (cf. to Fig. 2a). However, *E. coli* W_IPA exhibited a biomass yield of 0.33 g g^− 1^ for the growth phase compared to 0.18 g g^− 1^ for the knockout strains. *E. coli* W_IPA was also the only of the tested strains to produce significant amounts of isopropanol up to about 17.5 mmol L^− 1^ (Fig. 2), reaching an overall isopropanol yield of 0.45 Cmol Cmol^− 1^, which constitutes 89% of the carbon-balanced maximum theoretical yield (0.50 Cmol Cmol^− 1^). Moreover, onset of isopropanol production in *E. coli* W_IPA was observed only at the end of the growth phase, confirming a growth-decoupled production process found in a previous study investigating isopropanol production from acetate [32]. The strain also transiently formed acetate and pyruvate as side products, which were reassimilated once lactose was completely consumed.

In contrast, the knockout strains only produced marginal amounts of isopropanol and instead generated more pyruvate and acetate. The latter was subsequently not reassimilated. While pyruvate was taken up again after lactose was depleted, it was not directed towards isopropanol formation.

Since in *E. coli* W_KO2 (*ΔldhA ΔadhE*) and *E. coli* W_KO4 (*ΔldhA ΔadhE Δpta ΔfrdA*) the deleted enzymes are mainly located around the pyruvate and acetyl-CoA node, it seems plausible that isopropanol production is very sensitive to flux changes at these points. Interestingly, we did not observe any lactate or ethanol formation in the shake flask setting but the presence of *ldhA* and *adhE* appears to be vital for isopropanol production. Presumably, the cells use these routes to a small extent to dispose of surplus NADH.

Following the screening experiments, we used *E. coli* W_IPA for further bioreactor characterization.

### Aerobic cultivation on Whey

In a next step, the aim was to establish a bioreactor production process on whey. Therefore, *E. coli* W_IPA was cultivated aerobically on whey in a batch process (cf. to Supplementary Table 1, Supplementary Material 1), followed by a carbon-limited fed-batch phase with a specific growth rate of 0.1 h^− 1^. Based on the previous experiments, isopropanol production was expected to be low in such a scenario, since it had been shown to be growth-decoupled under aerobic conditions. The average biomass yield (0.3 g g^− 1^) necessary for the calculation of the feeding rate was obtained from preliminary batch cultivations (data not shown). In the batch phase, the cells consumed about 92 mmol L^− 1^ (31 g L^− 1^) lactose, 17 mmol L^− 1^ (3 g L^− 1^) galactose and 28 mmol L^− 1^ (2.5 g L^− 1^) lactate present in the medium and produced marginal amounts of acetate and about 60 mmol L^− 1^ (3.6 g L^− 1^) isopropanol. For both the batch and fed-batch phase, the carbon balance closed at about 112–113%, indicating carbon present in the sour whey that was not quantified prior to use, which was to be expected due to its complex nature. However, during the fed-batch phase, the overall isopropanol yield decreased to one tenth of the batch yield and the specific isopropanol formation rate showed a similar trend (Table 1). Instead of isopropanol, biomass formation was strongly favored.

We attributed this behavior to the competition between the product pathway and the TCA cycle at the acetyl-CoA node. During growth, ATP demand of cells is typically high, which can only be met if carbon enters the TCA cycle and ATP is either directly produced via substrate level phosphorylation or indirectly via NADH and respiration. If the carbon source is limiting, there is not enough acetyl-CoA to fuel the isopropanol pathway. In addition to carbon, the data suggests that the NADPH supply might be somewhat limited, since acetone was produced at a small rate during the fed-batch phase but not metabolized further to isopropanol (Table 1). Therefore, the carbon-limiting process strategy was not suited for isopropanol production due to the cells’ growth-decoupled behavior.

**Fig. 3:**
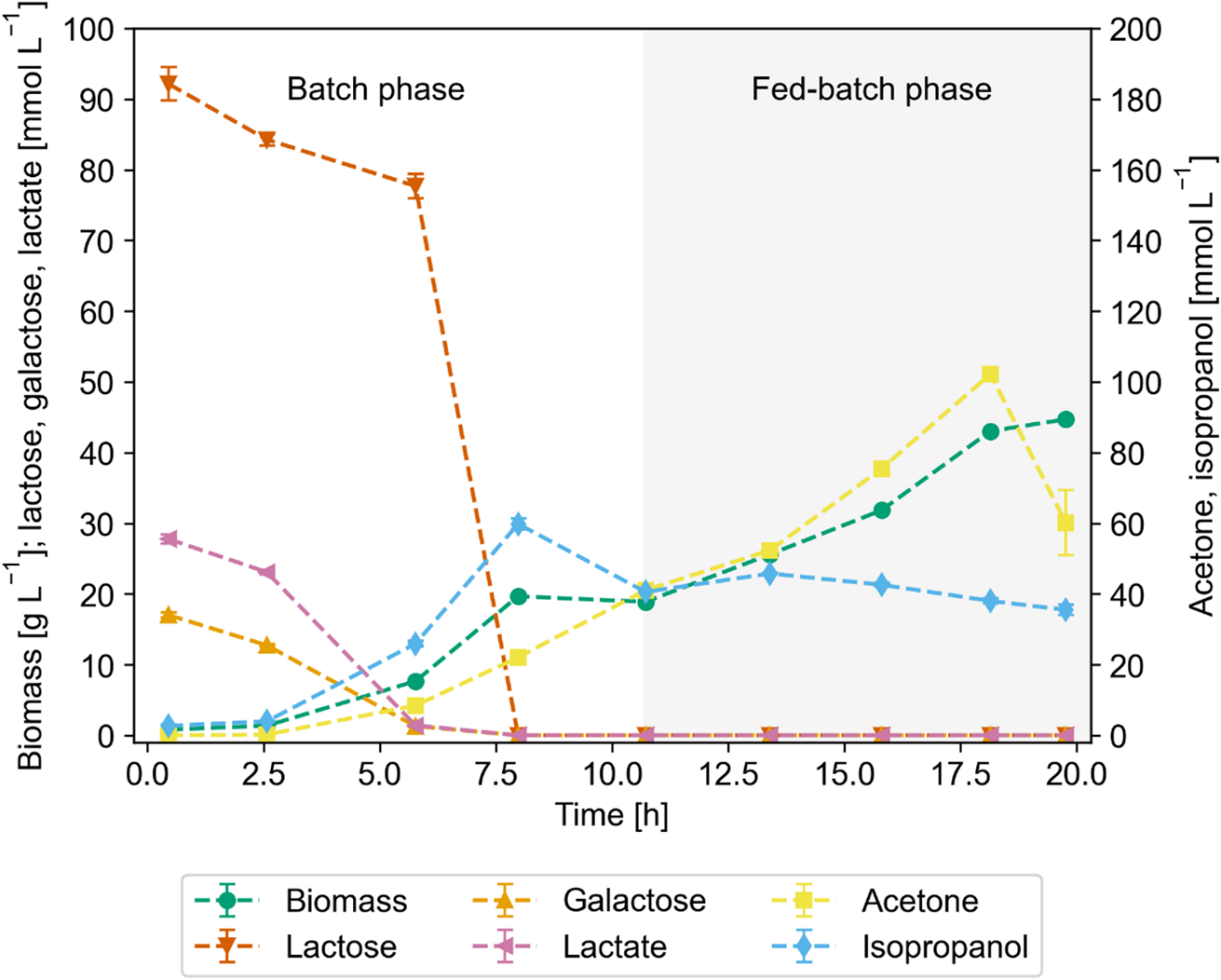
Concentration profiles during aerobic cultivation. Biomass, lactose, galactose, lactate, acetone, and isopropanol concentrations of isopropanol producing *E. coli* W in batch, fed-batch mode on whey

**Table 1:** Overall process parameters in aerobic conditions on Whey in a batch/fed-batch scenario with µ = 0.1 h^− 1^

Phase	Batch	Fed-batch
Y_IPA/LAC_ [Cmol Cmol^− 1^]	0.159 ± 0.001	0.015 ± 0.002
Y_ACO/LAC_ [Cmol Cmol^− 1^]	0.061 ± 0.002	0.058 ± 0.016
q_s, LAC_ [mmol g^− 1^ h^− 1^] (for the batch q_s, LACmax_ is given)	3.71 ± 0.6	0.648 ± 0.070
q_p, ipa_ [mmol g^− 1^ h^− 1^]	0.437 ± 0.020	0.031 ± 0.004
q_p, aco_ [mmol g^− 1^ h^− 1^]	0.168 ± 0.012	0.121 ± 0.034
Carbon balance [%]	112.6 ± 1.1	111.8 ± 3.1

### Establishing oxygen limiting conditions

Redesigning the process was necessary to limit growth to some extent but at the same time provide excess carbon to fuel the TCA cycle as well as the isopropanol pathway. Therefore, we considered a nutrient limitation as the tool to improve isopropanol production. However, since cheese whey is a complex substrate with various components and changing batch compositions, limitation strategies based on nitrogen or phosphorous limitation were not applicable (cf. to Supplementary Table 1, Supplementary Material 1).

The implementation of a microaerobic production process for alcohols (and other metabolites) has been described in several studies [24, 33]. Since the available oxygen can control growth and production (via NADH availability), oxygen limitation is a promising tool, especially for highly reduced products like alcohols. However, little is known on the exact oxygen uptake rates needed to sufficiently control production. Moreover, the gassing rate, the oxygen concentration in the ingas stream and the stirrer speed all influence the oxygen transfer to the medium and thereby the oxygen uptake rate. Consequently, definition and characterization of the working range of these parameters is a prerequisite in the context of an oxygen-limited bioprocess.

In a laboratory scale, a working volume of 1 L with a gassing rate of 60 L h^− 1^ (1 vvm) was chosen to reduce one of the variables. Subsequently, a 2D face centered DoE with 3 center points was designed with oxygen concentration and stirrer speed as variable parameters. An operating range for ingas oxygen between 10% and 30% and 400 to 1000 rpm for the stirrer speed were set. To avoid variations due to whey composition and preparation, the cells were grown on lactose as the model carbon source instead. Since it was not possible to measure O_2_-limitation directly with dissolved-oxygen-probes (DO-probes), microaerobic conditions were defined as starting from a dissolved oxygen (DO) content of 0%. All batches received the same amount of carbon source (batch + 1 pulse) and the processes continued until the carbon source was depleted.

**Fig. 4:**
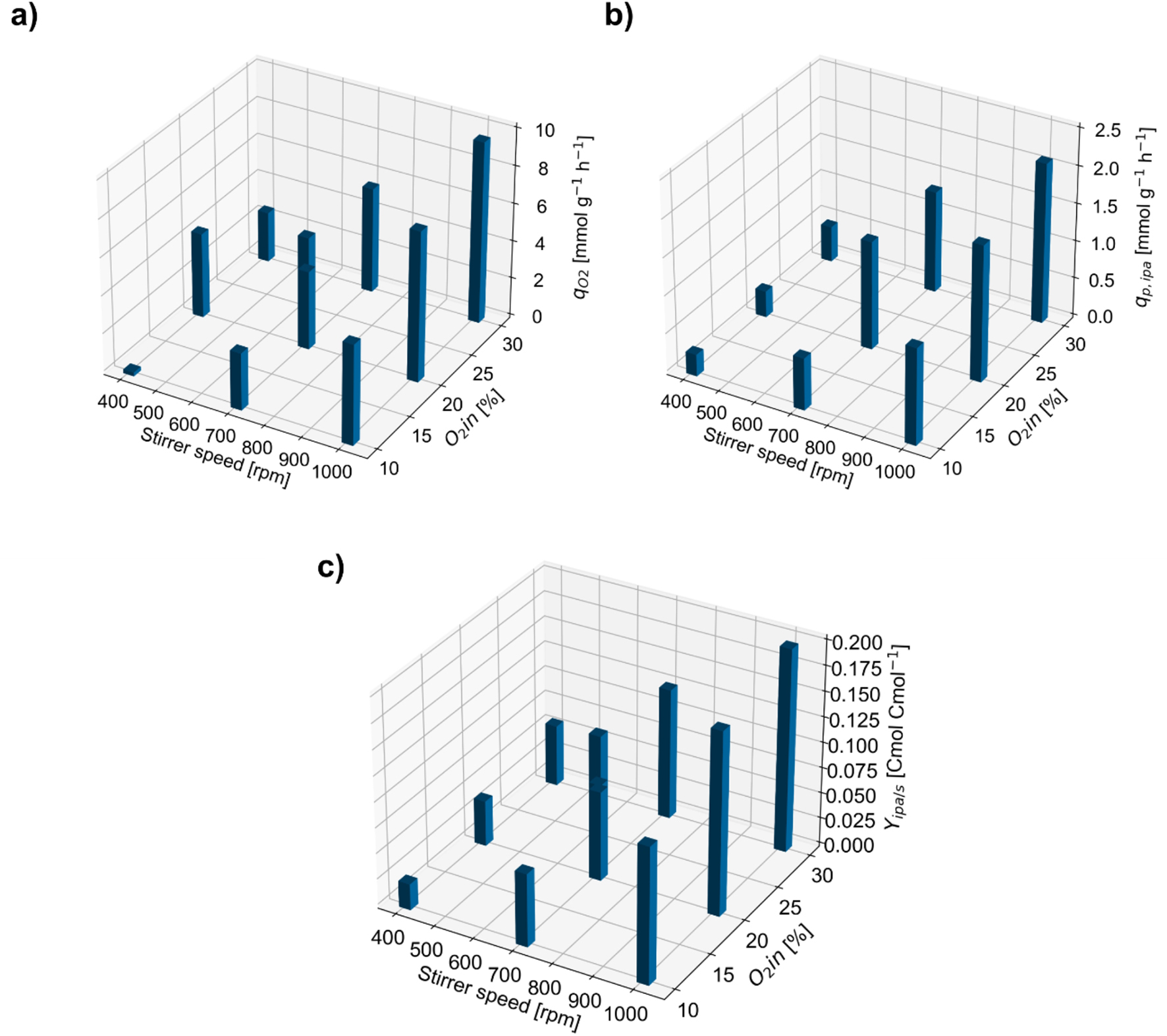
Specific parameters during microaerobic phases at the DoE setpoints. Oxygen uptake rates (q_O2_) (**a**), specific isopropanol production rates (q_p, ipa_) (**b**) and isopropanol yield per carbon of lactose (Y_ipa/s_)(**c**)

With this design, oxygen limitation set on early and lasted long for low-oxygen conditions (e.g. 48 h of microaerobic conditions at 400 rpm and 10% O_2__in) while for high-oxygen conditions the limitation was not as severe (e.g. 3 h of microaerobic conditions for 1,000 rpm and 30% O_2__in). Thus, different mean specific oxygen uptake rates (q_O2_) at the respective design points during the limitation phase were achieved, with low q_O2_ indicating a strongly microaerobic conditions and high q_O2_ corresponding to only mildly microaerobic conditions (Fig. 4a). As a result, different specific isopropanol formation rates (q_p, ipa_) and isopropanol yields (Y_ipa/s_) could be reached (Fig. 4b and c).

### Central carbon metabolism under oxygen limitation

**Fig. 5:**
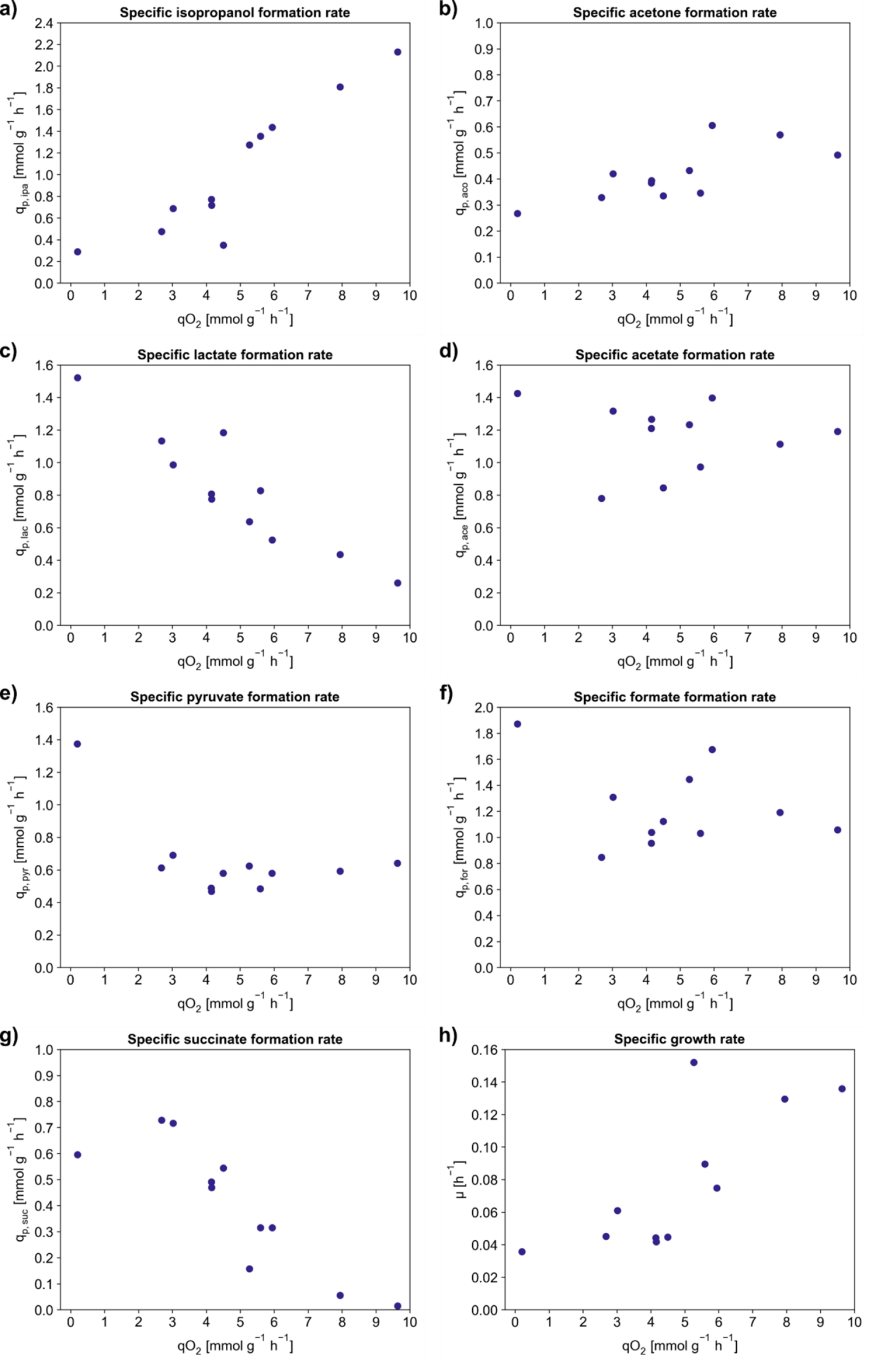
Influence of the specific oxygen uptake rate (q_O2_) on the specific metabolite formation rates. Isopropanol formation rate q_p, ipa_ (**a**), specific acetone formation rate q_p, aco_(**b**), specific lactate formation rate q_p, lac_ (**c**), specific acetate formation rate q_p, ace_ (**d**), specific pyruvate formation rate q_p, pyr_ (**e**), specific formate formation rate q_p, for_ (**f**), specific succinate formation rate q_p, suc_ (**g**), and specific growth rate µ (**h**) during microaerobic phases in the DoE experiments

In a next step, cell growth, product and side product formation were characterized and used for statistical data evaluation to gain insight into cellular metabolism under the different limiting conditions (cf. Section 2.9). Figure 5 shows the influence of the different microaerobic conditions on the mean specific formation rates of isopropanol (q_p, ipa_), acetone (q_p, aco_), lactate (q_p, lac_), acetate (q_p, ace_), pyruvate (q_p, pyr_), formate (q_p, for_), succinate (q_p, suc_) and the specific growth rate (µ). The results of the statistical data evaluation of the DoE are depicted in Supplementary Fig. 1–10, Supplementary Material 1.

The cells exhibited different mean q_O2_ in the oxygen limiting phases, ranging from 0.2 mmol g^− 1^ h^− 1^ for extremely limiting conditions to 9.6 mmol g^− 1^ h^− 1^ for only slightly limiting conditions. The specific rates for isopropanol and acetone formation as well as the specific growth rate were found to be directly proportional to the specific oxygen uptake (Fig. 5a, b and h, respectively). In contrast, the accumulation of lactate and succinate decreased with increasing q_O2_ (Fig. 5c and g). Pyruvate formation was constant at around 0.60 mmol g^− 1^ h^− 1^ for all tested conditions, except for the lowest q_O2_ observed where significantly more pyruvate was formed (Fig. 5e). Acetate and formate formation did not show a particular dependency on oxygen supply during microaerobic conditions. While there was a slight decrease in formate formation, acetate was produced regardless of the severity of the limitation (Fig. 5d and f). For the influence of q_O2_ on the acetone: isopropanol ratio (Y_ACO/IPA_) as well as on the specific lactose uptake rate, please cf. to Supplementary Fig. 11 and Supplementary Fig. 12, Supplementary Material 1. The specific lactose uptake rate remained between 2 and 3 mmol g^− 1^ h^− 1^ for all the tested microaerobic conditions.

Interestingly, during slightly microaerobic conditions higher specific oxygen uptakes could be reached than in the aerobic cultivations described in Sect. 3.2, which were kept at a constant dissolved oxygen level (dO_2_ of 20%). Under aerobic conditions, the mean q_O2_ never exceeded 6 mmol g^− 1^ h^− 1^ in the fed-batch phase and only briefly reached values around 9 mmol g^− 1^ h^− 1^ in the fed-batch phase (cf. to Supplementary Fig. 13, Supplementary Material 1). Collectively, the data suggest that a controlled increase of q_O2_ is vital for efficient isopropanol production. Concerning the influence of the stirrer speed or the ingas concentration, for most metabolites, stirrer speed is the more influential parameter (cf. to Supplementary Fig. 2–10, Supplementary Material 1). For the specific growth rate there was even a positive quadratic effect of the stirrer speed. Solely for the specific acetate and formate formation rates, the model coefficients indicate that instead the ingas oxygen concentration exerts a strongly negative effect.

The behavior of the pyruvate-formate lyase (PFL) and pyruvate dehydrogenase (PDH) responsible for pyruvate conversion into acetyl-CoA were somewhat unexpected. Typically, PDH is active under aerobic conditions and acitivity decreases as oxygen availability decreases due to inhibition by NADH [34]. In contrast, PFL is oxygen-sensitive, and therefore more active under oxygen-limiting or anaerobic conditions [35]. Due to these properties of PFL and PDH, it was anticipated that the specific oxygen uptake would have an impact on formate formation, since more carbon had to pass via PFL as PDH activity would decrease under strongly microaerobic conditions. However, the observed effect amounted only to a decrease of about 0.6 mmol g^− 1^ h^− 1^ of q_p, for_ (Fig. 5f). Even at very mild oxygen limiting conditions (q_O2_ at 9.6 mmol g^− 1^ h^− 1^), the PFL enzyme was still active and produced formate. On the other hand, pyruvate accumulation remained constant for most of the tested conditions. Therefore, it is likely, that residual PDH activity balances out the lower affinity of PFL for pyruvate at q_O2_ above 2 mmol g^− 1^ h^− 1^ (Fig. 5e). For PDH, a K_M_ value of 0.3 mM for pyruvate was reported, PFL exhibits an almost 7-fold higher K_M_ of 2 mM [36, 37].

For acetate, a negative correlation with the oxygen uptake rate has been previously reported which was based on a reduced transcription of citrate synthase (*gltA*) [38]. Moreover, it is known that NADH allosterically inhibits the GltA enzyme [39]. Nevertheless, even at a q_O2_ of 9.6 mmol g^− 1^ h^− 1^, the cells still exhibited specific acetate formation rates of 1.2 mmol g^− 1^ h^− 1^ (Fig. 5d). Given the complex nature of gene regulation for the transition from aerobic to anaerobic conditions, further research will be needed to characterize this observation and determine whether acetate formation under microaerobic conditions can be further reduced [40].

We also observed that the ratio of acetone-to-isopropanol ratio (Y_ACO/IPA_) increased for decreasing q_O2_ closer to anaerobic conditions (q_O2_ below 2 mmol g^− 1^ h^− 1^, cf. to Supplementary Fig. 11, Supplementary Material 1). We concluded that the most likely reason is an increasing shortage of NADPH, since the sources for NADPH, isocitrate dehydrogenase (ICDH) and malic enzyme (ME), are impaired under increasingly anaerobic conditions due to a reduced TCA cycle activity caused by GltA inhibition. It seems that the activity of the pentose phosphate pathway is insufficient to compensate this NADPH shortage.

### Critical points and ideal conditions for microaerobic isopropanol production

Our results point to three critical metabolic nodes for microaerobic isopropanol production on lactose. Firstly, the pyruvate node seems to be a major obstacle for carbon flux to isopropanol. A q_O2_ below 2 mmol g^− 1^ h^− 1^ causes increased accumulation of formate (1.87 mmol g^− 1^ h^− 1^), indicating more carbon passing through PFL. However, since pyruvate also accumulated at increased rates (1.37 mmol g^− 1^ h^− 1^) it seems likely that PFL is not efficient enough at converting pyruvate to acetyl-CoA. Secondly, due to limited oxygen availability under microaerobic conditions, there is a pseudo growth-coupled production of isopropanol (Figs. 5a and h, cf. to Supplementary Fig. 14, Supplementary Material 1). Thirdly, the NADPH supply is critical for shifting the ratio between acetone and isopropanol towards the latter.

Consequently, we identified mildly microaerobic conditions defined by a mean q_O2_ of 9.6 mmol g^− 1^ h^− 1^ as the best scenario for microaerobic isopropanol production on lactose.

### Confirmation runs on lactose and Whey

**Fig. 6:**
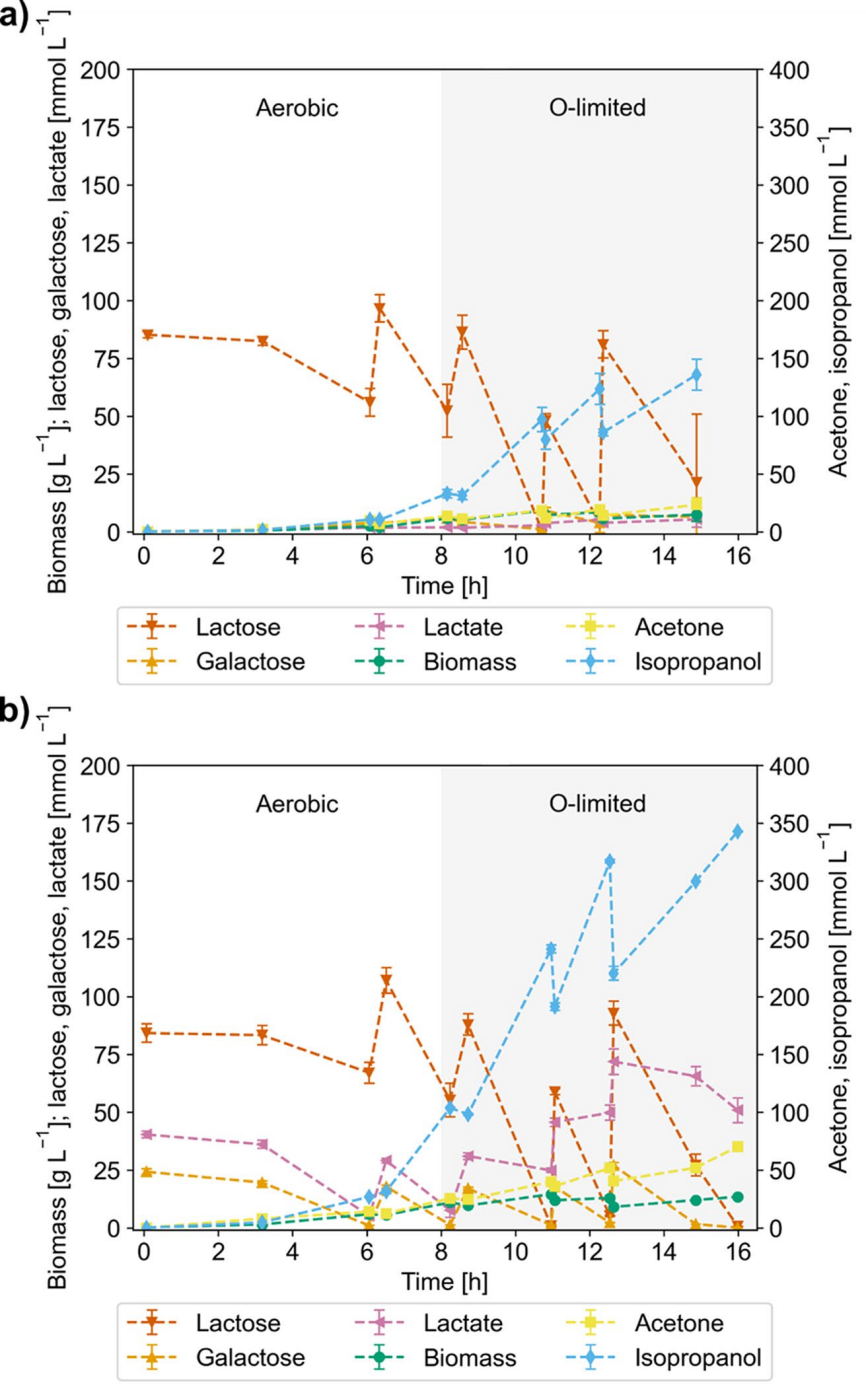
Confirmation runs at the best DoE conditions. Microaerobic conditions defined by 1000 rpm and 30% O_2_ in the ingas stream during, **a**) cultivation on lactose, **b**) cultivation on whey

To test the performance of the cells under the best conditions found in the DoE, confirmation runs in biological duplicates on lactose and on whey were conducted. Cultivations were carried out in batch and subsequent pulsed batch mode at a constant stirrer speed of 1000 rpm, an ingas O_2_ concentration of 30% and a gassing rate of 1 vvm. The results are depicted in Fig. 6 and the relevant physiological parameters are listed in Table 2. A statistical analysis of the specific isopropanol formation rate and the isopropanol yield on lactose and whey under aerobic and microaerobic conditions can be found in Supplementary Tables 2–5 in Supplementary Material 1.

**Table 2:** Overall process parameters in microaerobic conditions on lactose and Whey

	Lactose		Whey	
Phase	Aerobic	O-limited	Aerobic	O-limited
Y_IPA/LAC_ [Cmol Cmol^− 1^]	0.159 ± 0.015	0.256 ± 0.003	0.267 ± 0.004	0.309 ± 0.024
Y_IPA/LAC_ [mol mol^− 1^]	0.635 ± 0.061	1.02 ± 0.01	1.07 ± 0.01	1.23 ± 0.10
Y_ACO/LAC_ [Cmol Cmol^− 1^]	0.035 ± 0.004	0.042 ± 0.016	0.070 ± 0.006	0.050 ± 0.004
Y_ACO/LAC_ [mol mol^− 1^]	0.140 ± 0.014	0.167 ± 0.064	0.278 ± 0.023	0.200 ± 0.015
q_p, ipa_ [mmol g^− 1^ h^− 1^]	0.836 ± 0.035	2.757 ± 0.175	1.001 ± 0.020	2.215 ± 0.009
q_p, aco_ [mmol g^− 1^ h^− 1^]	0.399 ± 0.075	0.394 ± 0.113	0.261 ± 0.013	0.361 ± 0.001
Carbon balance [%]	108.2 ± 4.4	94.2 ± 4.9	123.8 ± 1.6	115.8 ± 4.8

Aerobic batch phase (dO2 » 1 %) and oxygen-limited (O-limited) pulsed batch scenario with a constant stirrer speed of 1,000 rpm and ingas oxygen concentration of 30 %

Table 2 legend. Aerobic batch phase (dO2 » 1%) and oxygen-limited (O-limited) pulsed batch scenario with a constant stirrer speed of 1,000 rpm and ingas oxygen concentration of 30%.

Overall, due to the slightly oxygen-limiting cultivation conditions, an efficient production of isopropanol on lactose and whey could be established reaching an overall space-time-yield of 0.54 g L^− 1^ h^− 1^ (8.99 mmol L^− 1^ h^− 1^) and 1.26 g L^− 1^ h^− 1^ (21.0 mmol L^− 1^ h^− 1^), respectively. Considering just the microaerobic phase, the volumetric isopropanol production rate (r_p, ipa_) amounts to 1.73 g L^− 1^ h^− 1^ (28.8 mmol L^− 1^ h^− 1^) and 2.44 g L^− 1^ h^− 1^ (40.6 mmol L^− 1^ h^− 1^) on lactose and whey respectively. The microaerobic process strategy yielded 135.8 ± 13.3 mmol L^− 1^ isopropanol after 15 h on lactose and 342.9 ± 0.4 mmol L^− 1^ isopropanol after 16 h on whey (Fig. 6). Compared to the aerobic cultivations with carbon limitation (cf. Table 1), this constitutes a 2.3-fold (lactose) and 5.7-fold (whey) increase in isopropanol titer. Without regulation of the dissolved oxygen concentration, already in the batch phase, the isopropanol yield was increased slightly in the microaerobic cultivations (Tables 1 and 2). In contrast to the yield in the carbon limited fed-batch of the aerobic cultivation (0.015 ± 0.002 Cmol Cmol^− 1^, cf. to Chap. 3.2), microaerobic conditions increased the isopropanol yield to 0.256 ± 0.003 Cmol Cmol^− 1^ on lactose and 0.309 ± 0.024 Cmol Cmol^− 1^ on whey, which constitute 51% and 62% of the carbon-balanced maximum theoretical yield of isopropanol on lactose (0.5 Cmol Cmol^− 1^ = 2 mol mol^− 1^). Interestingly, the acetone yield under microaerobic conditions was comparable to carbon-limiting conditions on whey.

In contrast to carbon-limiting cultivations, under microaerobic conditions cells showed a pseudo growth-coupled production behavior with a steady increase of biomass while isopropanol production was still supported even at an average specific growth rate of 0.084 ± 0.003 h^− 1^. Under carbon-limiting conditions (cf. to Chap. 3.2) with a controlled growth rate of about 0.100 h^− 1^, too little carbon was available for efficient isopropanol formation.

As we had already experienced for the aerobic cultivations, on lactose, the carbon balance was near 100% closed, while on whey, we found about 116–124% of carbon, indicating the presence of additional carbon that could not be exactly quantified due to the complex nature of whey.

### Further optimization of the process control and strain background

Quantitative assessment of microaerobic conditions revealed a direct correlation between q_O2_ and the specific isopropanol formation rate, enabling an effective process strategy to produce isopropanol from whey. However, there are several requirements with respect to process optimization before moving towards large-scale production.

To further enhance the isopropanol titer and space-time-yield to an economically viable range, additional investigations of the design space for microaerobic conditions might be required to identify the optimal q_O2_ for isopropanol production and to decrease by-product formation. Even at the least oxygen-limiting conditions (q_O2_ of 10 mmol g^− 1^ h^− 1^), by-product formation (acetate and pyruvate) was observed, suggesting there is carbon loss via by-products (e.g., pyruvate, acetate, and lactate). Especially the presence of pyruvate and lactate indicates a potential carbon bottleneck at the pyruvate node.

Therefore, further optimization of PDH and PFL could ensure more carbon being directed towards isopropanol formation. The data suggest that both PDH and PFL must be active to some degree at microaerobic conditions, but their combined activity is still not enough to avoid pyruvate accumulation. Consequently, increasing either PDH activity by making it less sensitive to NADH inhibition or improving PFL activity by developing a less oxygen-sensitive version may increase carbon throughput at the pyruvate node.

Furthermore, maintaining sufficient NADPH supply is crucial for isopropanol formation. Consequently, strain optimization by means of metabolic engineering might be needed to increase isopropanol yield and productivity. To increase flux from pyruvate to acetyl-CoA, PFL might be overexpressed. To ensure sufficient availability of redox equivalents (NADPH) for the conversion of acetone to isopropanol, the activity of the pentose phosphate pathway could be modified and/or additional transhydrogenases could be introduced [41, 42]. Alternatively, an NADPH-dependent formate dehydrogenase could be introduced. Combined with overexpression of PFL, acetyl-CoA and NADPH required for the isopropanol pathway can be provided in the correct stoichiometry.

Additionally, a suitable monitoring and control strategy is needed to maintain a q_O2_ in the desired range providing microaerobic conditions. While we were able to establish such conditions (and different mean q_O2_), it was not possible to directly control q_O2_. However, stringent control of q_O2_ will be paramount for any large-scale production process as q_O2_ regulates cell growth and the carbon allocation inside the cells (cf. to Figs. 4 and 5). To assess q_O2_ in real-time, information on the biomass concentration via on-line monitoring is needed in addition to the volumetric oxygen uptake rate OUR obtained from off-gas analysis. The information on the physiological parameter q_O2_ may then be used in a feedback control loop to control process variables affecting oxygen transfer to the culture (e.g. stirrer speed, gassing rate, partial pressure of O_2_ in ingas).

Owing to the pseudo-growth coupled conditions, continuous production of isopropanol might be possible, either in a one-step conversion operating in chemostat mode (with or without cell retention to boost productivity) or as a two-stage process. In the first stage, biomass could be continuously produced and transferred to the second stage operated at microaerobic conditions enabled by on-line biomass monitoring and q_O2_-control to favor isopropanol production.

## Conclusions

In this study an efficient bioprocess for isopropanol production from lactose and whey using engineered *E. coli* W was established. A quantitative assessment of microaerobic conditions revealed correlations of q_O2_ and isopropanol formation (positive) and by-product formation (negative), providing fundamental insights into intracellular carbon fluxes under oxygen-limiting conditions. Moreover, microaerobic conditions enabled pseudo growth-coupled isopropanol production. Strain optimization with the focus on improving precursor and redox equivalent availability as well as establishing tight oxygen and biomass monitoring and control were identified as crucial parameters for process optimization. Collectively, this study forms a sound basis toward sustainable, large-scale isopropanol production.

## Electronic supplementary material

Below is the link to the electronic supplementary material.

Supplementary Material 1

## Data Availability

The authors declare that the data supporting the findings of this study are available within the paper and its supplementary files. Should any raw data files be needed in another format they are available from the corresponding author upon reasonable request.
