# Supplementary material for: Improving sustainable isopropanol production in engineered Escherichia coli W via oxygen limitation
Source: Microb Cell Fact. 2025 Apr 26;24:94. doi: 10.1186/s12934-025-02720-1 (PMC12032697; doi:10.1186/s12934-025-02720-1)
Supplement: Supplementary file 1 — Supplementary Material 1 [file 12934_2025_2720_MOESM1_ESM.docx]

**Additional File 1**

# Whey composition

The whey concentrate used for the cultivations had the composition described in Supplementary Table 1.

Supplementary Table 1. Sour whey concentrate composition.

| Lactose [g L^-1^] | 200 |
| --- | --- |
| Galactose [g L^-1^] | 22 |
| Lactate [g L^-1^] | 4.5 |
| Glutamate [mg L^-1^] | 165 |
| Ammonia [mmol L^-1^] | 20.4 |
| Phosphate [mmol L^-1^] | 93.4 |

The concentrations of lactose, galactose and lactate were confirmed by HPLC measurements. The concentrations of glutamate, ammonia and phosphate were determined via Cedex Bio HT Analyzer (Roche, Switzerland) kits.

# Design of Experiment plots

- 1. Contour plots

Supplementary Figure 1 shows the contour plots of the DoE after statistical data analysis.


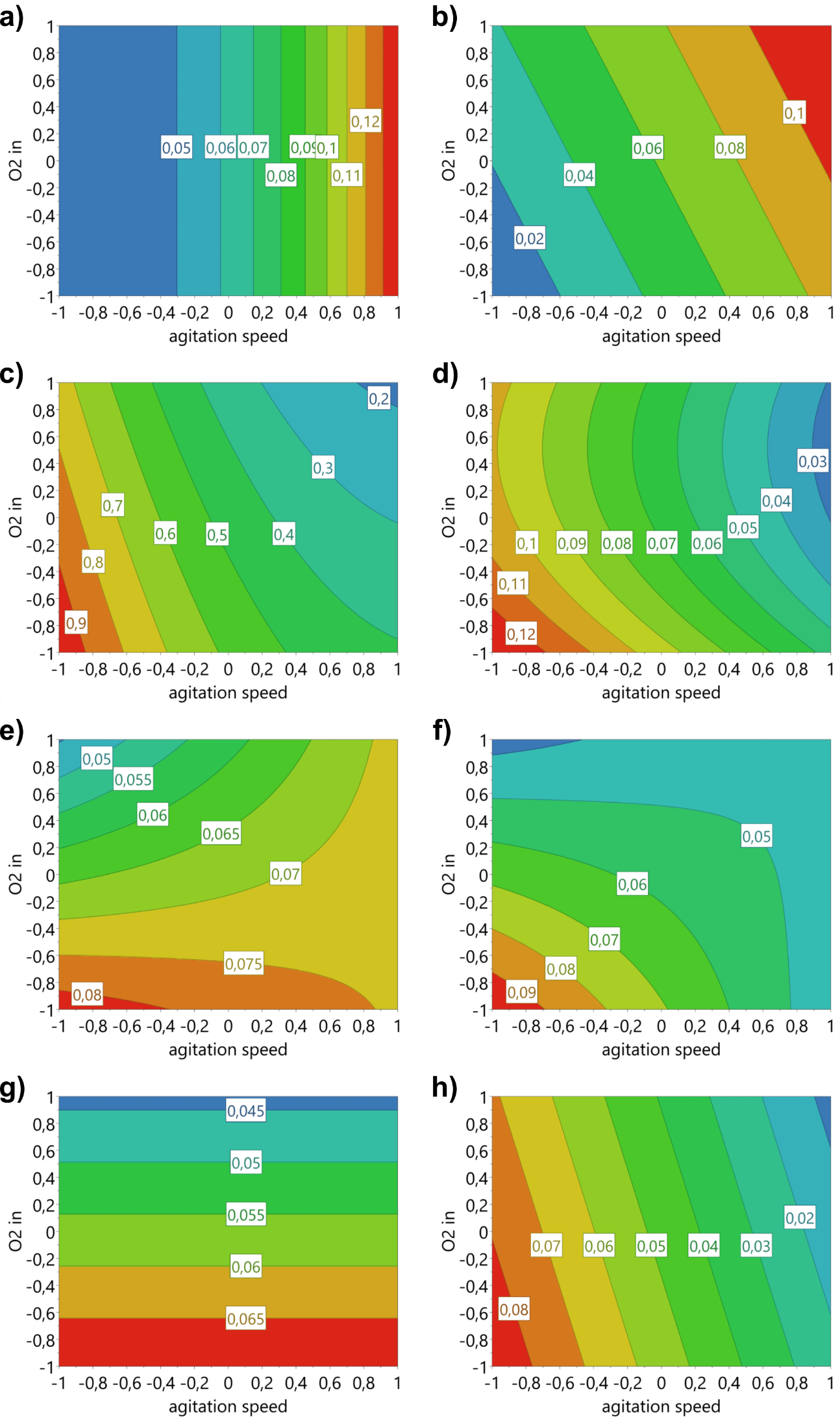


Supplementary Figure 1. Contour plots of the specific growth rate (a), specific isopropanol formation rate [g g^-1^ h^-1^] (b), ratio between acetone and isopropanol (c), specific lactate formation rate [g g^-1^ h^-1^] (d), specific acetate formation rate [g g^-1^ h^-1^] (e), specific pyruvate formation rate [g g^-1^ h^-1^] (f), specific formate formation rate [g g^-1^ h^-1^] (g), and specific succinate formation rate [g g^-1^ h^-1^] (h) during microaerobic phases in the DoE experiments.

Supplementary Figure 2 to Supplementary Figure 10 show the coefficient plots of the most important parameters during the DoE.


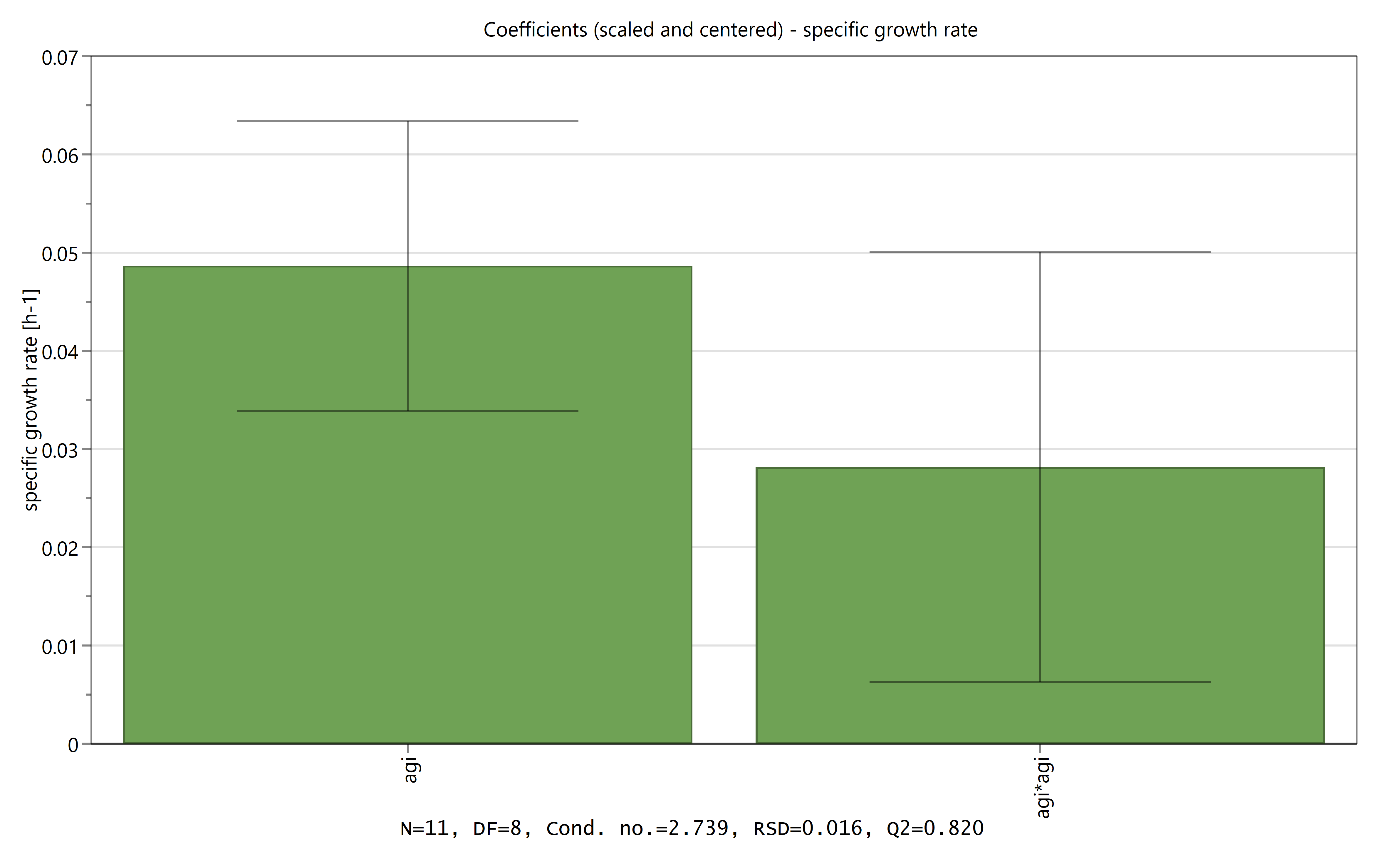


Supplementary Figure 2. Model coefficients for the specific growth rate.


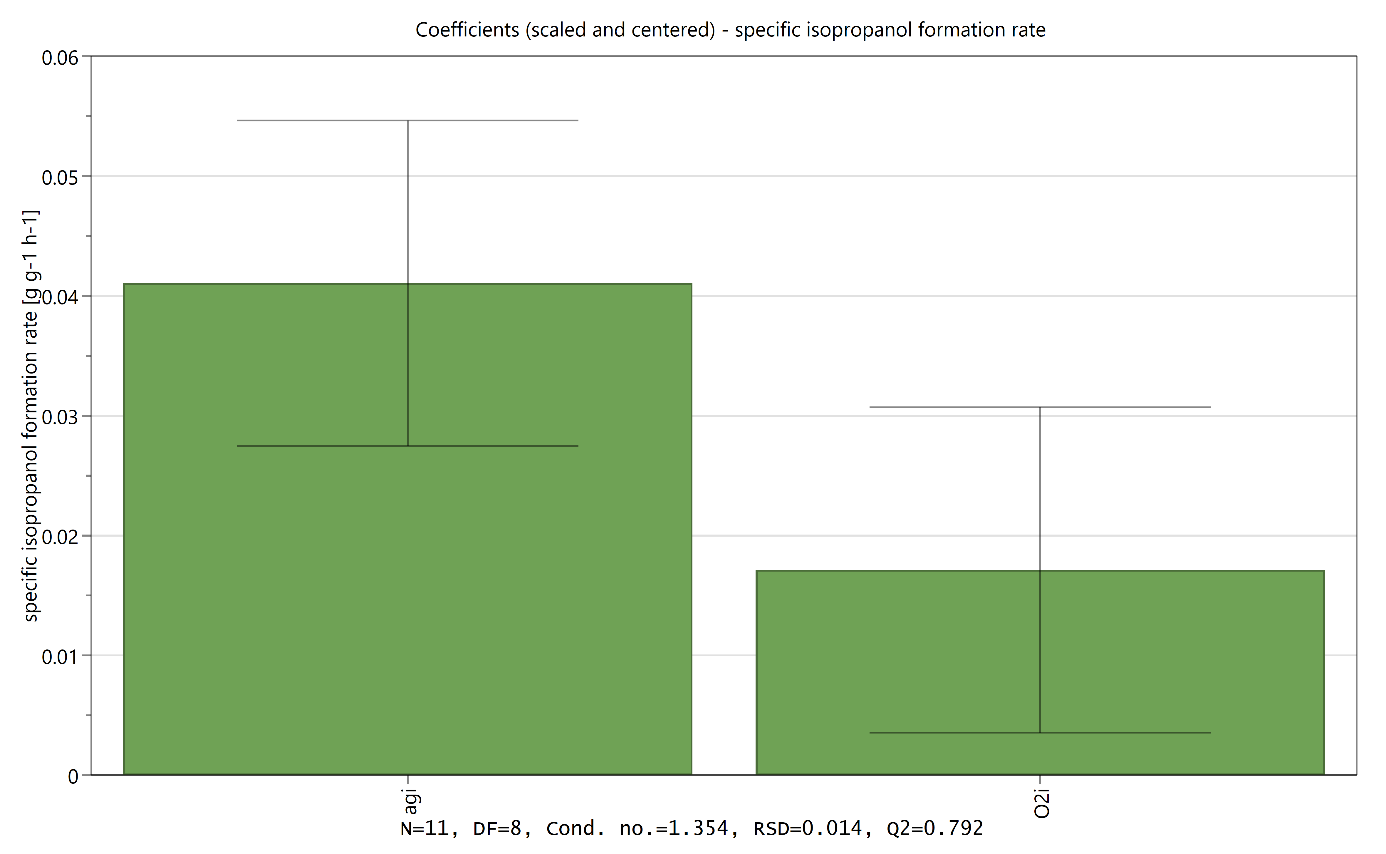


Supplementary Figure 3. Model coefficients for the specific isopropanol formation rate.


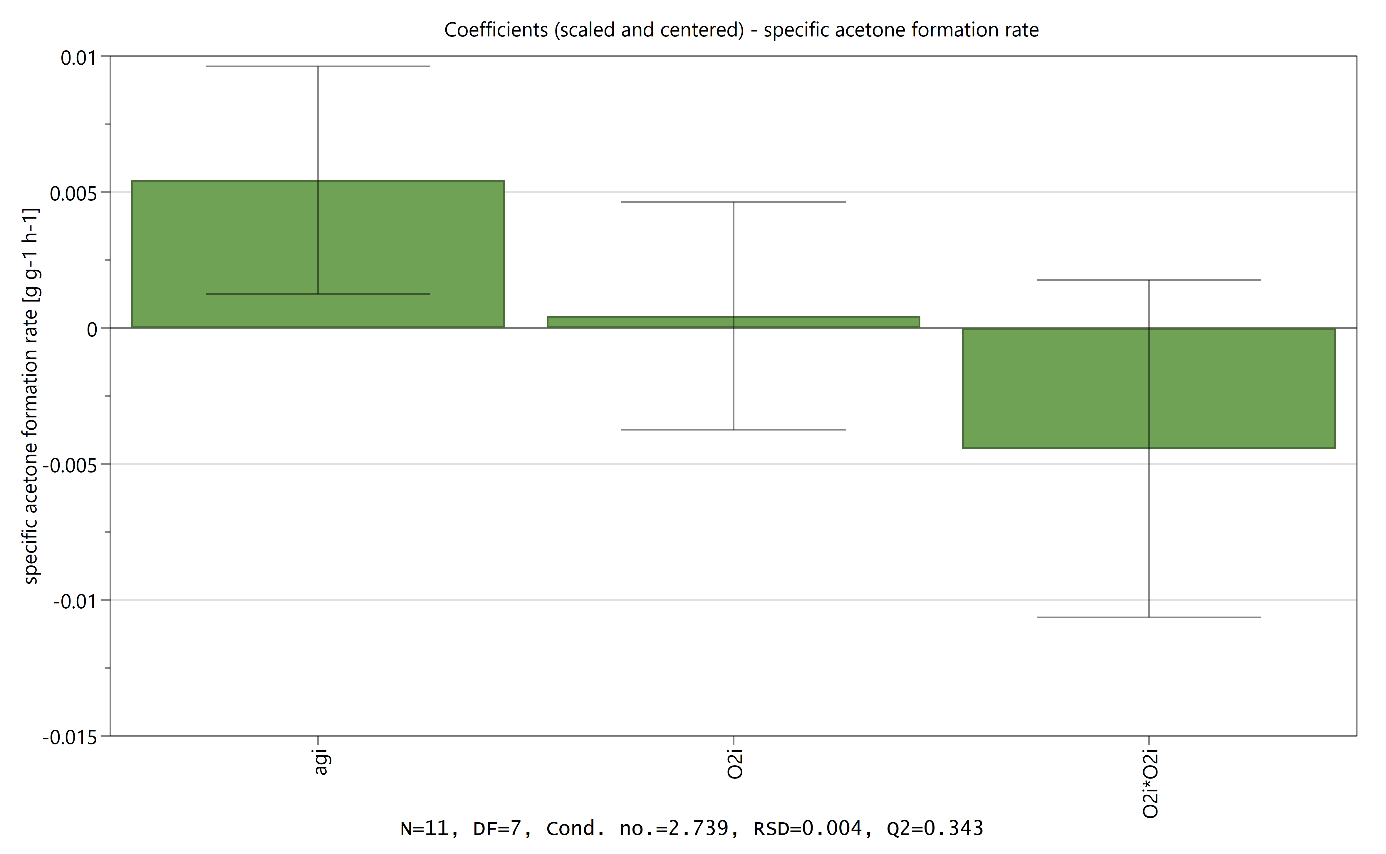


Supplementary Figure 4. Model coefficients for the specific acetone formation rate.


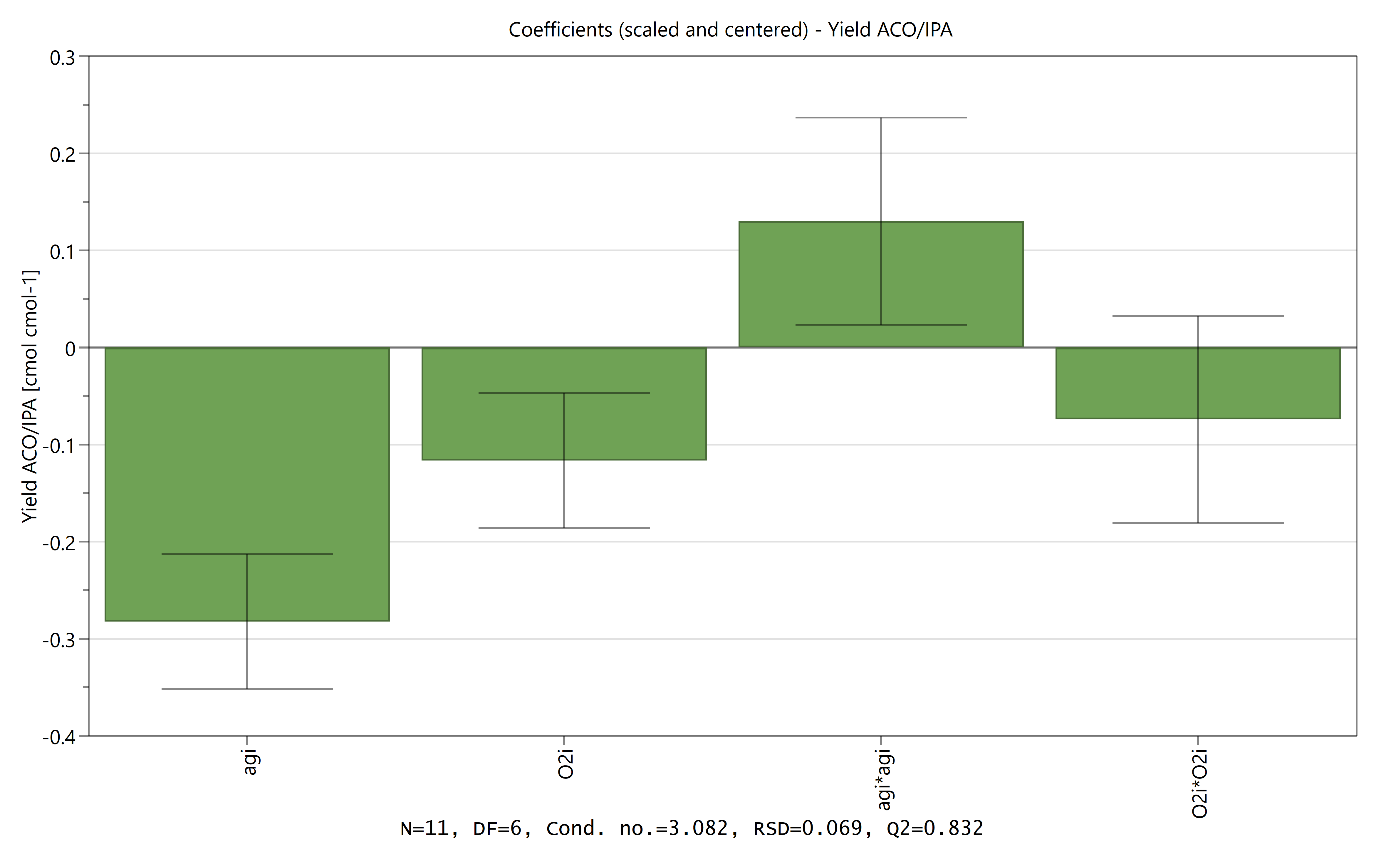


Supplementary Figure 5. Model coefficients for the ratio between acetone and isopropanol.


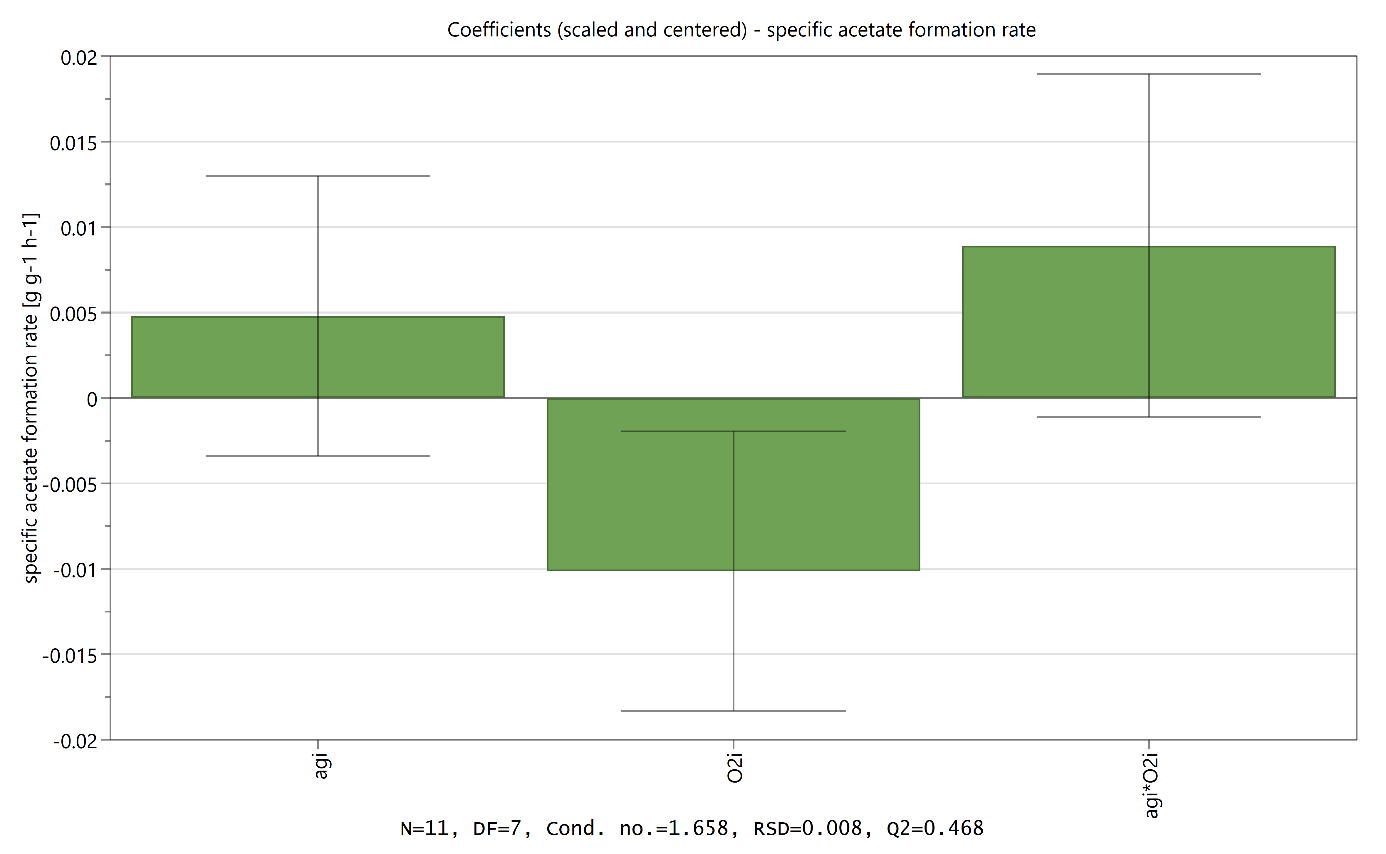


Supplementary Figure 6. Model coefficients for the specific acetate formation rate.


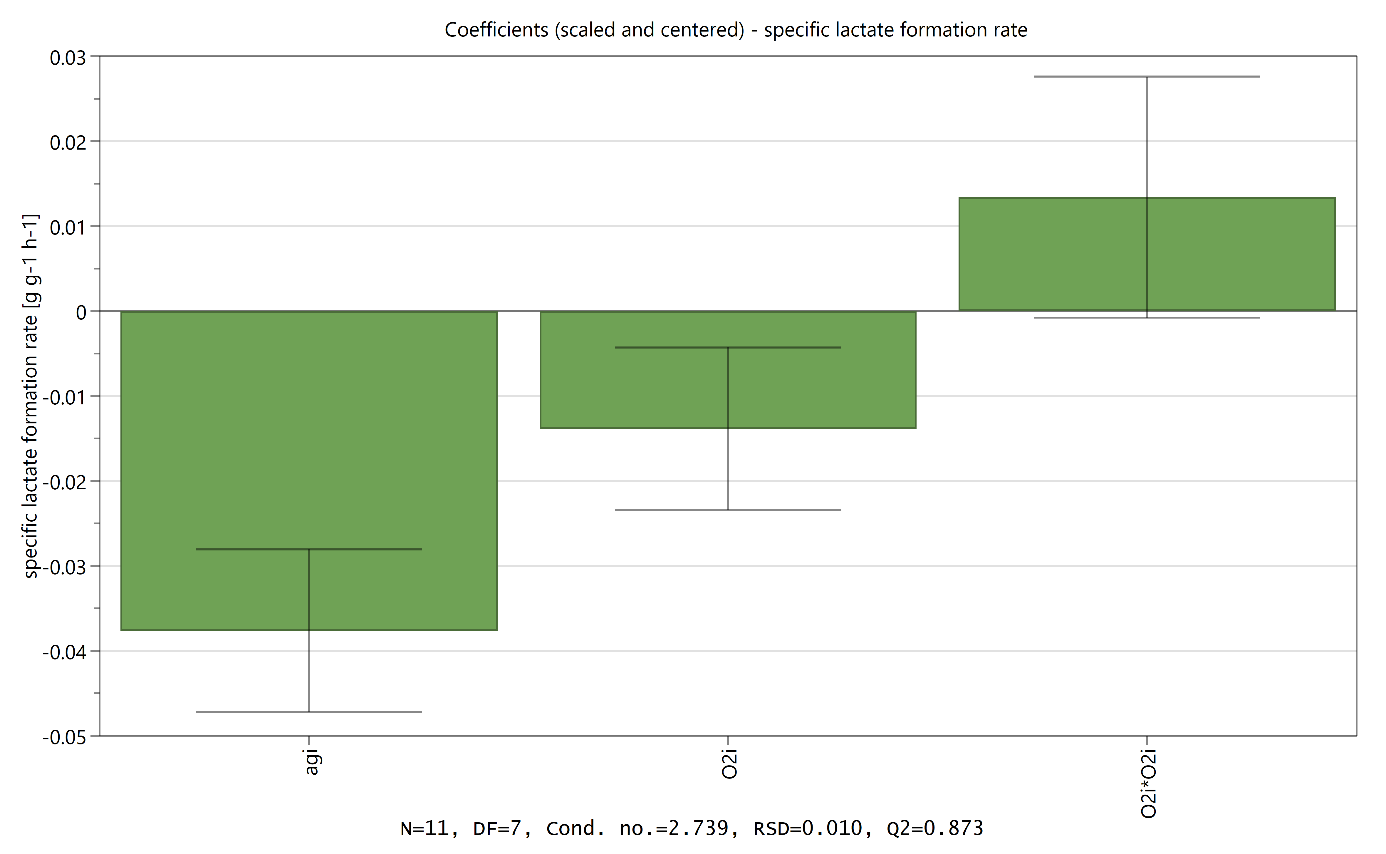


Supplementary Figure 7. Model coefficients for the specific lactate formation rate.


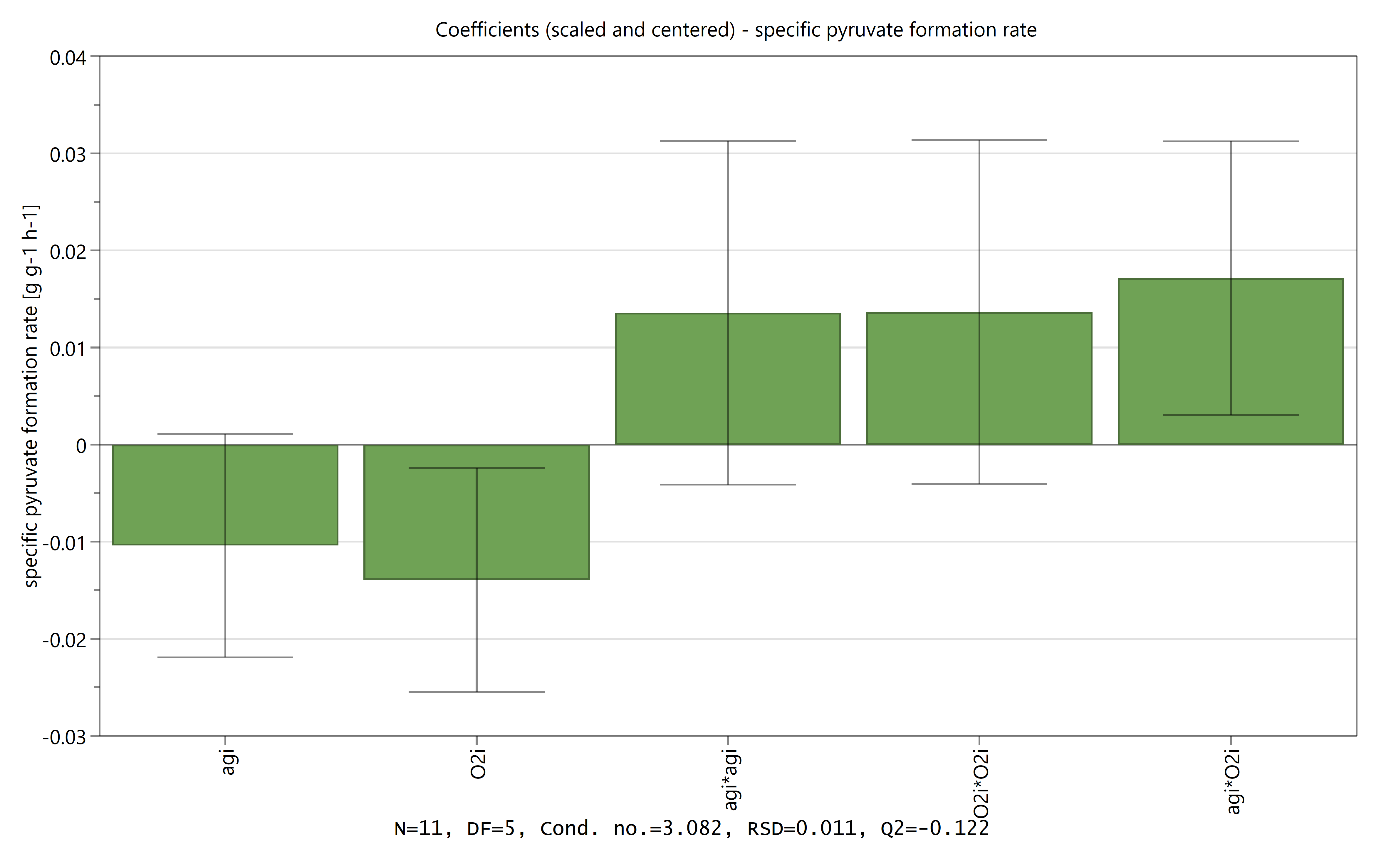


Supplementary Figure 8. Model coefficients for the specific pyruvate formation rate.


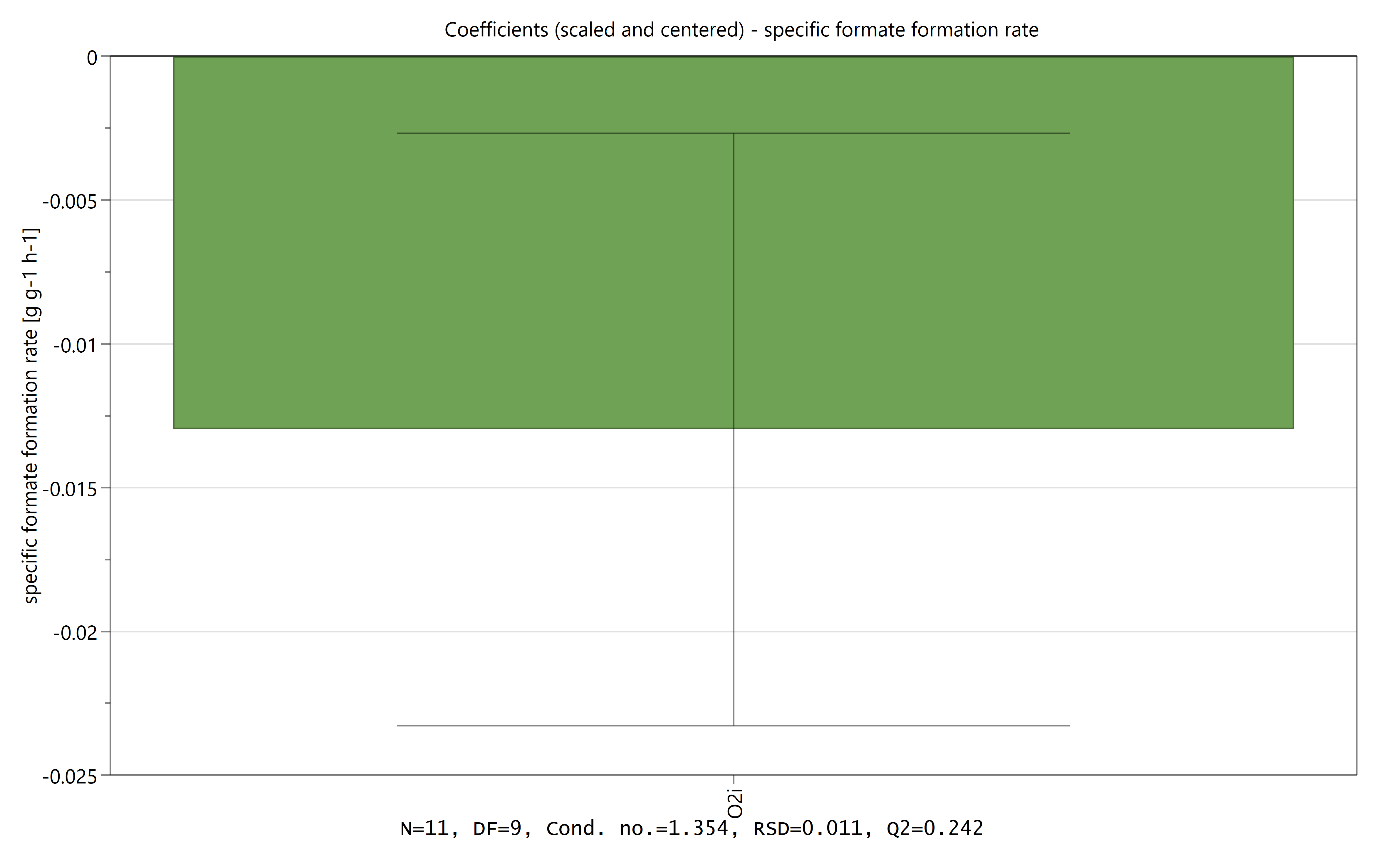


Supplementary Figure 9. Model coefficients for the specific formate formation rate.


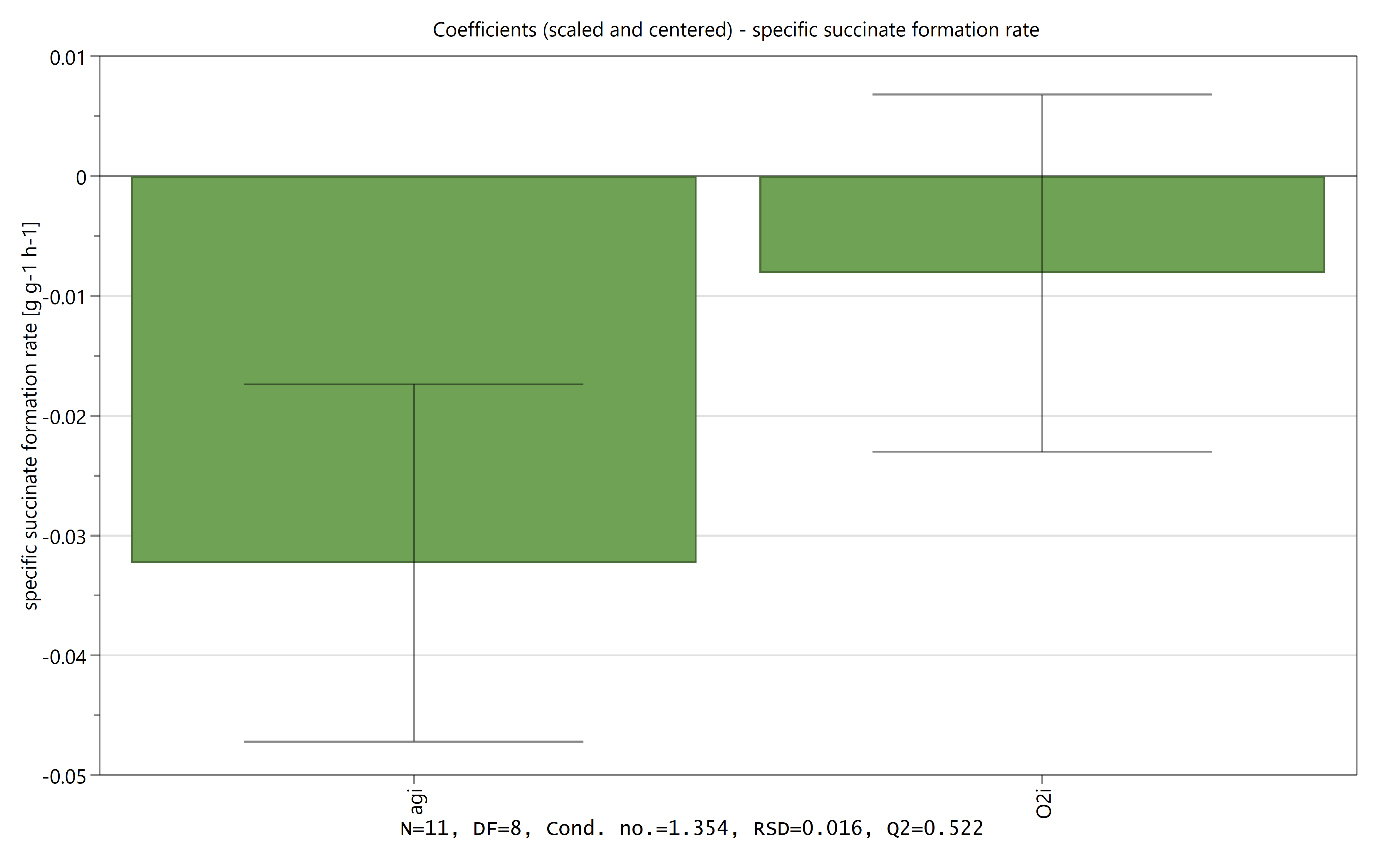


Supplementary Figure 10. Model coefficients for the specific succinate formation rate.

# Additional data at microaerobic conditions

The dependency of the ratio between acetone and isopropanol formed on q_O2_ is depicted in Supplementary Figure 11, while the dependency of the specific lactose uptake rate on qO2 is shown in Supplementary Figure 12.

Supplementary Figure 11. Ratio between acetone and isopropanol (Y_ACO/IPA_) in relation to the mean specific oxygen uptake rate (q_O2_).

Supplementary Figure 12. Specific lactose uptake rate in relation to the mean specific oxygen uptake rate (q_O2_)

# Aerobic cultivations specific oxygen uptake rate

The specific oxygen uptake rate of an exemplary aerobic fed-batch cultivation (dO_2_ controlled at 20 %) is depicted in Supplementary Figure 13.

Supplementary Figure 13. Specific oxygen uptake rate over time during an exemplary aerobic fed-batch cultivation.

# Pseudo growth coupled behavior

Depicting the specific isopropanol formation rate during microaerobic conditions as function of the specific growth rate revealed the pseudo-growth coupled production in Supplementary Figure 14.

Supplementary Figure 14. Specific isopropanol formation rate qp,ipa as function of the specific growth rate (µ) during microaerobic conditions.

# Statistical analysis

The results of the t-tests for the specific isopropanol formation rates and the isopropanol yields on lactose and whey under aerobic and microaeobic conditions are depicted in Supplementary Table 2 – 5.

Supplementary Table 2. t-test result for q_p,ipa_ on lactose between aerobic (variable 1) and microaerobic (variable 2) conditions.

| *Lactose q_p,ipa_* | *Variable 1* | *Variable 2* |
| --- | --- | --- |
| Mean | 0.8363362 | 2.75698519 |
| Variance | 0.00124493 | 0.0306687 |
| Observations | 2 | 2 |
| Pooled variance | 0.01595682 |  |
| Hypothetical difference of means | 0 |  |
| Degrees of freedom (df) | 2 |  |
| t-statistic | -15.204596 |  |
| **P(T<=t) one-sided** | **0.00214889** |  |
| Critical t-value for one sided t-test | 2.91998558 |  |

Supplementary Table 3. t-test result for q_p,ipa_ on whey between aerobic (variable 1) and microaerobic (variable 2) conditions.

| *Whey q_p,ipa_* | *Variable 1* | *Variable 2* |
| --- | --- | --- |
| Mean | 1.00141587 | 2.21471493 |
| Variance | 0.00038359 | 8.8491E-05 |
| Observations | 2 | 2 |
| Pooled variance | 0.00023604 |  |
| Hypothetical difference of means | 0 |  |
| Degrees of freedom (df) | 2 |  |
| t-statistic | -78.972372 |  |
| **P(T<=t) one-sided** | **8.0152E-05** |  |
| Critical t-value for one sided t-test | 2.91998558 |  |

Supplementary Table 4. t-test result for Y_ipa/lac_ on lactose between aerobic (variable 1) and microaerobic (variable 2) conditions.

| *Lactose Y_ipa/lac_* | *Variable 1* | *Variable 2* |
| --- | --- | --- |
| Mean | 0.15883538 | 0.25578778 |
| Variance | 0.00023574 | 1.0021E-05 |
| Observations | 2 | 2 |
| Pooled variance | 0.00012288 |  |
| Hypothetical difference of means | 0 |  |
| Degrees of freedom (df) | 2 |  |
| t-statistic | -8.7460655 |  |
| **P(T<=t) one-sided** | **0.00641104** |  |
| Critical t-value for one sided t-test | 2.91998558 |  |

Supplementary Table 5. t-test result for Y_ipa/lac_ on whey between aerobic (variable 1) and microaerobic (variable 2) conditions.

| *Whey Y_ipa/lac_* | *Variable 1* | *Variable 2* |
| --- | --- | --- |
| Mean | 0.266977 | 0.30856017 |
| Variance | 1.2621E-05 | 0.00057489 |
| Observations | 2 | 2 |
| Pooled variance | 0.00029376 |  |
| Hypothetical difference of means | 0 |  |
| Degrees of freedom (df) | 2 |  |
| t-statistic | -2.4261862 |  |
| **P(T<=t) one-sided** | **0.0680283** |  |
| Critical t-value for one sided t-test | 2.91998558 |  |

The results of the t-tests for the metabolites in the shake flask experiments at the end of the cultivation are shown in Supplementary Table 6 – 14.

Supplementary Table 6. t-test result for c_ipa_end_ for E. coli W and E. coli W KO2

| *c_ipa_end_* | *E. coli* W | *E. coli* W KO2 |
| --- | --- | --- |
| Mean | 0.971766667 | 0.206733333 |
| Variance | 0.022839223 | 0.071280863 |
| Observations | 3 | 3 |
| Pooled variance | 0.047060043 |  |
| Hypothetical difference of means | 0 |  |
| Degrees of freedom (df) | 4 |  |
| t-statistic | 4.31916531 |  |
| **P(T<=t) one-sided** | **0.006227803** |  |
| Critical t-value for one sided t-test | 2.131846786 |  |

Supplementary Table 7. t-test result for c_ipa_end_ for E. coli W and E. coli W KO4

| *c_ipa_end_* | *E. coli* W | *E. coli* W KO4 |
| --- | --- | --- |
| Mean | 0.971766667 | 0.049466667 |
| Variance | 0.022839223 | 1.03333E-07 |
| Observations | 3 | 3 |
| Hypothetical difference of means | 0 |  |
| Degrees of freedom (df) | 2 |  |
| t-statistic | 10.5703943 |  |
| **P(T<=t) one-sided** | **0.004415751** |  |
| Critical t-value for one sided t-test | 2.91998558 |  |

Supplementary Table 8. t-test result for c_ipa_end_ for E. coli W KO2 and E. coli W KO4

| *c_ipa_end_* | *E. coli* W KO2 | *E. coli* W KO4 |
| --- | --- | --- |
| Mean | 0.206733333 | 0.049466667 |
| Variance | 0.071280863 | 1.03333E-07 |
| Observations | 3 | 3 |
| Hypothetical difference of means | 0 |  |
| Degrees of freedom (df) | 2 |  |
| t-statistic | 1.020259198 |  |
| **P(T<=t) one-sided** | **0.207465296** |  |
| Critical t-value for one sided t-test | 2.91998558 |  |

Supplementary Table 9. t-test result for c_ace_end_ for E. coli W and E. coli W KO2

| *c_ace_end_* | *E. coli* W | *E. coli* W KO2 |
| --- | --- | --- |
| Mean | 0.023733333 | 4.4856 |
| Variance | 0.001689813 | 0.02822688 |
| Observations | 3 | 2 |
| Pooled variance | 0.010535502 |  |
| Hypothetical difference of means | 0 |  |
| Degrees of freedom (df) | 3 |  |
| t-statistic | -47.6189253 |  |
| **P(T<=t) one-sided** | **1.01956E-05** |  |
| Critical t-value for one sided t-test | 2.353363435 |  |

Supplementary Table 10. t-test result for c_ace_end_ for E. coli W and E. coli W KO4

| *c_ace_end_* | *E. coli* W | *E. coli* W KO4 |
| --- | --- | --- |
| Mean | 0.023733333 | 2.819033333 |
| Variance | 0.001689813 | 0.028769293 |
| Observations | 3 | 3 |
| Pooled variance | 0.015229553 |  |
| Hypothetical difference of means | 0 |  |
| Degrees of freedom (df) | 4 |  |
| t-statistic | -27.74153392 |  |
| **P(T<=t) one-sided** | **5.02165E-06** |  |
| Critical t-value for one sided t-test | 2.131846786 |  |

Supplementary Table 11. t-test result for c_ace_end_ for E. coli W KO2 and E. coli W KO4

| *c_ace_end_* | *E. coli* W KO2 | *E. coli* W KO4 |
| --- | --- | --- |
| Mean | 4.4856 | 2.819033333 |
| Variance | 0.02822688 | 0.028769293 |
| Observations | 2 | 3 |
| Pooled variance | 0.028588489 |  |
| Hypothetical difference of means | 0 |  |
| Degrees of freedom (df) | 3 |  |
| t-statistic | 10.79736329 |  |
| **P(T<=t) one-sided** | **0.000849644** |  |
| Critical t-value for one sided t-test | 2.353363435 |  |

Supplementary Table 12. t-test result for c_pyr_high_ for E. coli W and E. coli W KO2

| *c_pyr_high_* | *E. coli* W | *E. coli* W KO2 |
| --- | --- | --- |
| Mean | 1.633166667 | 2.280566667 |
| Variance | 0.024878573 | 0.916414043 |
| Observations | 3 | 3 |
| Hypothetical difference of means | 0 |  |
| Degrees of freedom (df) | 2 |  |
| t-statistic | -1.155768879 |  |
| **P(T<=t) one-sided** | **0.183596785** |  |
| Critical t-value for one sided t-test | 2.91998558 |  |

Supplementary Table 13. t-test result for c_pyr_high_ for E. coli W and E. coli W KO4

| *c_pyr_high_* | *E. coli* W | *E. coli* W KO4 |
| --- | --- | --- |
| Mean | 1.633166667 | 3.6609 |
| Variance | 0.024878573 | 0.00323271 |
| Observations | 3 | 3 |
| Pooled variance | 0.014055642 |  |
| Hypothetical difference of means | 0 |  |
| Degrees of freedom (df) | 4 |  |
| t-statistic | -20.94744851 |  |
| **P(T<=t) one-sided** | **1.53471E-05** |  |
| Critical t-value for one sided t-test | 2.131846786 |  |

Supplementary Table 14. t-test result for c_pyr_high_ for E. coli W KO2 and E. coli W KO4

| *c_pyr_high_* | *E. coli* W KO2 | *E. coli* W KO4 |
| --- | --- | --- |
| Mean | 2.280566667 | 3.6609 |
| Variance | 0.916414043 | 0.00323271 |
| Observations | 3 | 3 |
| Hypothetical difference of means | 0 |  |
| Degrees of freedom (df) | 2 |  |
| t-statistic | -2.493067758 |  |
| **P(T<=t) one-sided** | **0.065099329** |  |
| Critical t-value for one sided t-test | 2.91998558 |  |
